# Exploring Communication Dynamics Between Patients and Healthcare Providers in Oncology: A Systematic Review

**DOI:** 10.1111/hex.70519

**Published:** 2025-12-11

**Authors:** Pauline Justin, Valentyn Fournier, Ambre Naeyaert, Lisa Laroussi‐Libeault, Christelle Duprez, Pascal Antoine, Kristopher Lamore

**Affiliations:** ^1^ Univ. Lille, CNRS, UMR 9193 – SCALab – Sciences Cognitives et Sciences Affectives Lille France

**Keywords:** cancer, communication, healthcare professionals, patient, systematic review

## Abstract

**Objective:**

Patient–physician communication in oncology has been studied extensively. Most studies have focused on healthcare professionals' (HCPs) communication, showing the importance of a patient‐centred approach. While some studies have explored the behaviours of patients and their relatives, the majority have centred on HCPs' behaviours, with much less attention to patients' communication patterns during consultations. The main objective of this systematic review was to examine how patients communicate and behave during oncology consultations and to identify the factors influencing these behaviours.

**Methods:**

Five databases were searched to find studies analyzing the communication patterns of patients and their relatives during oncology consultations that were audio‐ and/or video‐recorded.

**Results:**

Based on the 34,779 references identified, we included 47 studies in our review. Three main themes emerged from the analysis: (1) patients' communication patterns and the topics discussed during oncology consultations; (2) factors influencing patient communication; and (3) patients' perception of and satisfaction with the consultation.

**Conclusions:**

Patients exhibited active behaviours during consultations. However, many factors can influence interactions. We recommend taking a comprehensive approach that involves considering communication factors and supporting the development of patient‐centred strategies tailored to individual patient needs. HCPs should not only practice patient‐centred care; they should also implement specific actions to address patients' psychosocial needs. Future research should also utilize complex models to better understand the dynamics of patient–provider communication.

**Abbreviations**
HCP: healthcare professional
MMAT: mixed‐methods appraisal tool
PCC: patient‐centred communication
PRISMA: Preferred Reporting Items for Systematic Reviews and Meta‐Analyses
PRO: patient‐reported outcomes
SWiM: synthesis without meta‐analysis

## 1 Introduction

Patient–physician communication in oncology is extensively studied. However, most research has centred on healthcare professionals' (HCPs) communication, consistently underscoring the importance of adopting a patient‐centred approach [1, 2, 3, 4]. Patient‐centred communication (PCC) involves HCPs seeking out the patient's perspective, encouraging expression, asking questions and considering patients' verbal input, emotions and psychosocial needs [3, 5]. This approach enhances patients' satisfaction with care [6], treatment adherence [1, 7], comfort in expressing emotions [8], active participation by asking more questions [8] and information sharing [9]. As a result, patients obtain more information from physicians, who are more likely to consider their needs [10]. PCC increases patients' knowledge [9, 11, 12], participation in care [13], reduces anxiety [14] and improves quality of life [15, 16].

However, despite efforts to implement PCC, some communication needs, especially emotional needs, go unmet [17]. While a systematic review has highlighted communication with HCPs as a primary need in supportive care [18], PCC is not considered the gold standard for consultations, and it can be challenging to implement. Contextual factors can also influence communicative quality. For instance, at some stages of cancer, it can be difficult to accommodate the patient's perspective, especially when extensive medical information must be shared [19, 20, 21]. In addition, while some patients feel that they do not have enough time with their doctors [18], studies have shown that the quality of communication does not improve with an increase in the duration of the consultation [22]. Moreover, individual factors can affect communication, such as a perceived lack of competence of providers in communicating an emotional response to a patient's emotional difficulties [23]. The presence of a family member can also affect what role the patient takes on during consultations [24], with relatives often asking more questions than patients during consultations [25]. Simultaneously, patient‐related factors can affect the type of consultations. For example, some patients prefer not to be involved in consultations or medical decisions [26, 27], so they appreciate doctor‐centred communication [28, 29]. These preferences can be related to demographic characteristics, such as educational level and culture [30, 31]. Finally, relational factors such as trust in one's medical practitioner can influence patients' [17] and relatives' behaviours [25] during consultations. For instance, some patients and their relatives may not feel comfortable spontaneously and explicitly sharing their emotions with their physicians [32, 33]. Moreover, anxiety and distress can inhibit patients' expression of emotions or asking of questions [30, 34, 35]. The literature has suggested that patient involvement in consultations may fluctuate, requiring professionals to adapt thereto [28].

Although studies have mainly focused on verbal communication to demonstrate patient involvement in medical consultations, studies have also shown that patients often communicate their concerns in different ways during consultations. Patients can express their emotions, such as worries, through questions or verbal and nonverbal cues [14]. However, physicians do not always recognize such cues [14, 36], which may be due to a lack of specific training and skills [37]. In Abu‐Odah et al.'s [4] systematic review, the main barriers to PCC included a lack of knowledge and training on communication among HCPs.

Therefore, training programmes aimed at improving HCPs' communication skills have been developed to promote their practice of PCC [38, 39] since training physicians on communication skills can result in patients more actively participating in consultations [19, 40]. This is because trained physicians ask more open‐ended questions, inviting patients to ask their own questions and share their concerns [19, 41, 42]. They also display more empathy and are more sensitive and attentive to patients' words and cues [41, 42, 43, 44]. However, the literature has shown that such training is not prioritized among HCPs: training requires time, which physicians often lack [45].

Significant gaps remain in our understanding of how patients communicate during consultations, particularly in regard to nonverbal and interactional dynamics. While previous research has mostly analyzed verbal exchanges, nonverbal communication and broader interaction patterns remain underexplored [46, 47]. Patient communication and involvement are influenced by multiple factors, including individual, relational and contextual factors. However, these factors have often been studied in isolation, so their combined influence on communication during oncology consultations has been explored insufficiently.

Therefore, the aim of this systematic review is to synthesize existing evidence for patient communication during oncology consultations. Specifically, we examine (1) how patients communicate and behave with HCPs, focusing on both verbal and nonverbal cues, and (2) the factors influencing communication and patient involvement in this interaction, based exclusively on audio or video recordings of consultations.

## 2 Materials and Methods

In this systematic review, we followed the Preferred Reporting Items for Systematic Reviews and Meta‐Analyses (PRISMA) [48], and this study is registered on PROSPERO (CRD42023414983).

### 2.1 Search Strategy

Five electronic databases were used for this systematic review, which was conducted on 7 June 2023 (updated on 22 April 2025): Embase, PsycArticles, PsycInfo, PubMed and Web of Science. An additional search was performed on Google Scholar and Google Web Search. Our search was restricted to scientific articles written in English, French or Spanish. The databases were explored with a combination of keywords relating to cancer patients' and their relatives' communication during consultations with HCPs (see Box 1).

Box 1. **Database search terms**

(‘communicat*’ or ‘verbal’ or ‘talk’)

**AND**

(‘patient*’ or ‘carer*’ or ‘caregiv*’ or ‘relative*’ or ‘famil*’ or ‘partner*’ or ‘couple*’ or ‘spouse*’)

**AND**

(‘cancer’ or ‘oncology’ or ‘neoplasm’ or ‘leukaemia’ or ‘lymphoma’ or ‘myeloma’ or ‘sarcoma’)

**NOT**

(‘paediatric’ or ‘childhood’ or ‘end‐of‐life’ or ‘palliative care’ or ‘review’ or ‘meta‐analysis’)

### 2.2 Selection Criteria

To be included, the studies had to meet SPIDER criteria [49]: They had to include adult cancer patients at any point of the cancer continuum, excluding paediatric and end‐of‐life situations, and they could include relatives but could not focus exclusively on them (Sample). They had to report patients' verbal or nonverbal communication behaviours during consultations (Phenomenon of Interest). They could be observational or interventional, provided they included observations of consultations and, specifically, behaviours in real time (Design), which were recorded as audio or video media (Evaluation). Finally, they had to use a qualitative, quantitative or mixed‐method design (Research Type).

Studies were excluded if they only focused on relatives or HCPs' communication, did not address patient's communication behaviours, or were case reports, protocols, reviews, systematic or meta‐analyses (see Table 1).

**Table 1:** Criteria for study eligibility.

Inclusion criteria	Exclusion criteria
– Samples comprising adult cancer patients at any point of the cancer continuum – Studies that include relatives but do not focus exclusively on them – Studies providing results for patients' verbal or nonverbal communication behaviours during consultations – Observational or interventional studies with audio or video recordings of consultations – Studies following qualitative, quantitative, or mixed‐method designs	– Samples comprising patients with paediatric cancers – Samples comprising patients in end‐of‐life situations – Studies focusing only on relatives' or healthcare professionals' communication – Studies lacking an assessment of patients' communication behaviours – Case reports, protocols, reviews, or systematic or meta‐analyses

### 2.3 Study Selection and Data Extraction

We performed our study selection using the web application Covidence (available at www.covidence.org). After removing all duplicates, two of the four researchers (P.J., V.F., K.L. and A.N.) independently screened the studies based on their titles and abstracts. Then, a full‐text review of the remaining studies was conducted to identify eligible studies. If disagreements arose between the researchers, a consensus was reached by consulting the study coordinator (K.L.). Data extracted (checked by P.J. and V.F.) included country, funding, research design and methods, purpose, participant characteristics, measures and results.

### 2.4 Quality Assessment

The methodological quality of the included studies was assessed using the Mixed‐Methods Appraisal Tool (MMAT) [50, 51, 52]. This checklist of seven categories evaluates and describes mixed studies and reviews, including qualitative, quantitative and mixed‐methods studies. Two of the three researchers (P.J., V.F. and A.I.) independently assessed the methodological quality of each study. If disagreements arose, the evaluation was discussed to reach a consensus.

### 2.5 Synthesis Methods

Given the heterogeneity in study designs, interventions and reported outcomes, a narrative synthesis was conducted following synthesis without meta‐analysis (SWiM) reporting guidelines [53], which gave us a transparent and systematic framework for synthesizing evidence since a quantitative meta‐analysis was not feasible. Guided by a structured data extraction process (see Section 2.3), studies were compared according to shared design and outcome measure characteristics. This approach enabled us to identify recurring patterns and obtain a broad understanding of trends and associations across studies.

## 3 Results

### 3.1 Study Selection

**Figure 1:**
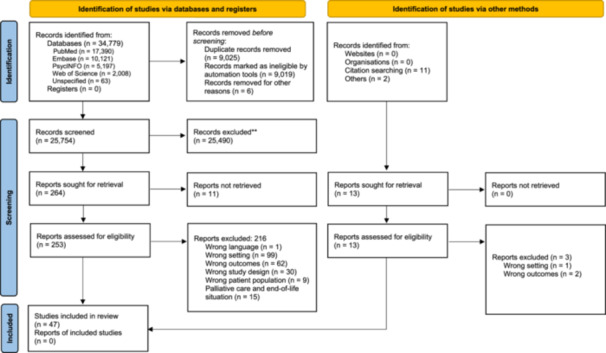
PRISMA flowchart.

Among 34,779 database results and 13 manually identified articles, 253 studies were identified as potentially eligible. After applying our selection criteria, 216 studies were excluded, and 47 studies were kept for analysis (see the flow diagram in Figure 1).

### 3.2 Study Characteristics

Studies were published between 2000 and 2025. Studies were conducted in several countries: 25 studies (53.19%) were conducted in North America, 19 in Europe (40.43%), 2 in Asia (4.26%) and 1 in Australia (2.28%). Moreover, 16 studies (34.04%) used a mixed‐methods design, 10 (21.28%) were randomized controlled trials, 10 (21.28%) used a qualitative design, 9 (19.15%) used a quantitative design and 1 (2.28%) was a non‐randomized trial. To retrieve patients' communication behaviours, 38 studies (80.85%) used audio‐recorded consultations and 9 (19.15%) utilized video‐recorded consultations. Most of the included studies (82.98%) were conducted solely with doctors as HCPs, and only 14 (29.79%) included both patients and relatives. An extensive presentation of the characteristics of the studies is presented in Supporting Information 1.

### 3.3 Quality Assessment of the Included Studies

According to MMAT criteria, most qualitative studies were of high quality; only one study had a limitation in how findings were derived from the data [54]. Randomized controlled trials showed variability in methodological rigour. While some criteria were addressed thoroughly, including randomization [15, 55, 56, 57, 58], the baseline comparability of groups [15, 57, 59], outcome completeness [15, 46, 57, 59] and blinding [15, 25, 46, 55, 57, 59, 60], participants' adherence [57, 59] indicated potential risks of bias, warranting a more cautious interpretation of their results. The only non‐randomized study [47] achieved the high quality for most criteria, although how the authors accounted for the confounders was unclear, suggesting the need for some caution. Quantitative descriptive studies largely met quality criteria, but limitations in their sampling strategy [61, 62, 63], sample representativeness [61, 63, 64, 65, 66] and nonresponse bias [14, 24, 61, 63, 67] reduced confidence in the generalization of their findings. Mixed‐methods studies generally addressed the divergences between quantitative and qualitative results adequately. Several studies had unclear or unmet criteria related to the rationale for choosing a mixed‐methods design [68, 69, 70, 71] and adherence [10, 19, 57, 62, 67, 68, 70, 72, 73, 74, 75, 76], to methodological standards in each tradition. Further details about the MMAT analysis that we conducted are presented in Supporting Information 2.

The analysis of the included studies revealed three major themes: (1) patient communication patterns and the topics discussed during oncology consultations, (2) factors influencing patient communication, and (3) patient perceptions of and satisfaction with consultations.

### 3.4 Theme 1: Patients' Communication Patterns and the Topics Discussed During Oncology Consultations

Thirty‐three articles explored patients' interactions with HCPs during oncology consultations [10, 13, 14, 15, 19, 25, 46, 55, 57, 59, 61, 62, 63, 64, 65, 66, 68, 69, 70, 71, 72, 73, 74, 77, 78, 79, 80, 81, 82, 83, 84, 85, 86]. Four sub‐themes were identified: (1) topics discussed by patients during the consultation, (2) verbal and proactive communication among patients, (3) indirect and/or nonverbal communication among patients, and (4) withdrawal and passivity among some patients.

#### 3.4.1 Topics Discussed by Patients During Their Consultations

As found in the reviewed studies, during medical consultations, many topics are discussed. Twenty‐two included articles described these topics in detail [10, 13, 19, 25, 46, 55, 57, 61, 62, 63, 64, 66, 68, 69, 72, 73, 74, 77, 80, 82, 83], explaining that consultative conversations mainly focus on cancer treatment, medical procedures and potential side‐effects [10, 13, 14, 19, 25, 46, 55, 63, 64, 66, 72, 73, 74, 77, 80, 85]; the nature of the cancer itself [10, 13, 62, 66, 77, 82]; the causes of the cancer [77]; diagnosis, prognosis, survival chances or the risk of recurrence [19, 25, 46, 63, 66, 71, 72, 73, 74, 77, 80, 85]. Logistical and administrative aspects are also mentioned [10, 19, 25, 63, 77, 82, 85, 86], with patients wanting to know the costs and procedures related to their health insurance [72, 77], as well as the number of appointments they would need so that they can plan accordingly [10, 77]. Patients also often express the need for additional information or reassurance about their condition and choices [46, 66].

Patients also addressed psychosocial aspects related to their cancer, particularly those related to their quality of life [82]. Only a few studies discussed these subjects in detail [64], while others noted that these topics are often only briefly mentioned [63, 69, 71, 80]. However, Mitchell et al. [13], in their longitudinal study, showed that the topics discussed during medical consultations between patients with colorectal cancer and HCPs (including oncologists and nurses) evolve over time: While they found that initial consultations focus more on biomedical information, subsequent consultations increasingly include discourse on the patient's psychosocial needs. Several studies [13, 61, 63, 69, 71, 83] also noted that patients do discuss their experiences and concerns, especially in relation to treatment side‐effects, but they do so rarely. Finally, patients' opinions and preferences are addressed [46, 62, 66, 68, 74], particularly to facilitate shared medical decision‐making [68, 74]. This heterogeneity of topics discussed seems to align with the variety of needs that patients express [63], with biomedical aspects being prioritized [13, 14, 62, 82] and limited attention being paid to psychosocial aspects [62, 63, 69, 82].

#### 3.4.2 Verbal and Proactive Communication Among Patients

Twenty‐two studies explored patient involvement during consultations. Despite the relatively short duration of consultations, generally not exceeding 30 min [14, 63, 64, 65, 66, 75, 82], patients usually communicate verbally and proactively [10, 13, 19, 25, 57, 59, 61, 63, 64, 65, 66, 68, 71, 74, 76, 79, 80, 81, 82, 83, 84], managing this time to ask questions, gather information and ask clarification [10, 13, 14, 19, 25, 66, 68, 71, 76, 79, 80, 81, 82]. Several studies reported that patients ask an average of 20 questions per consultation [19, 25, 66, 68, 82, 85], using these opportunities to clarify information [71, 80, 84] or start new conversations [10, 57, 59, 64, 84]. Patients also often mention their symptoms, describe their problems, their concerns or confusion and discuss their experiences related to the illness [13, 59, 61, 63, 71, 80, 83]. Studies classified these patients as proactive and highly involved in their care [74, 76, 79, 81]. Some patients were also described as direct, using explicit terms and assertive communication [81, 84], while others use methods such as keeping a diary to list their symptoms and concerns [63] or preparing a list of questions to present to their physicians [76].

#### 3.4.3 Indirect and/or Nonverbal Communication Among Patients

Eleven studies reported that patients use indirect and nonverbal communication [15, 19, 62, 66, 69, 72, 73, 78, 81, 84, 87]. This indirect communication includes the use of metaphors, allusions to some topics without addressing them directly, or comments made in passing [73]. Other patients take micro‐pauses or do not finish their statements, indicating hesitation, uncertainty, or overwhelming emotions [78], while some talk about their emotions and concerns indirectly while discussing medical information [66, 71, 73, 84, 87]. Furthermore, nonverbal communication occurs through facial expressions [15, 66, 73], crying, sighing, laughter or emotional incongruence [15, 62, 69, 71, 73, 78].

#### 3.4.4 Withdrawal and Passivity Among Some Patients

Some patients are considered more passive or withdrawn [10, 13, 46, 59, 63, 65, 74, 76, 78, 80, 82, 83] since they do not ask any questions but only listen to the information provided by the physician [13, 46, 74, 80, 82], simply responding to the questions they are asked [76] or nodding to signify agreement [10, 65]. Other patients show no concern, assuring physicians that they are fine [63] or denying their symptoms when asked about them [59, 83]. Finally, some patients do not wish to be involved in decisions about their health and try to defer decision‐making to the HCP [78]. These varied communication patterns are not fixed but are influenced by multiple factors. In the following section, we explore how patient, physician and contextual characteristics shape communication during consultations.

### 3.5 Theme 2: Factors Influencing Patient Communication

Patient involvement during consultations can be influenced by many factors, as reported in 33 of our included studies. These factors can be categorized into four sub‐themes: (1) patients' sociodemographic characteristics, (2) patients' health status and consultation‐related factors, (3) physicians' influence, and (4) the role of relatives.

#### 3.5.1 Patients' Sociodemographic Characteristics

Patient involvement, including active participation, number of questions asked, or expressed signals, depends on various individual and sociodemographic characteristics [19, 25, 56, 58, 61, 65, 68, 69, 75, 81, 88, 89]. Age plays a significant role in active participation [14, 25, 56, 58, 61, 65, 69, 85, 88], with younger patients tending to ask more questions and be more active during consultations than older patients [14]. Gender was also mentioned in four studies [14, 65, 67, 75], indicating that women tend to talk more than men and ask more questions [14, 75]. Physicians were also found to be more likely to provide greater explanations to women [65] and focus conversations with women on psychosocial rather than solely medical aspects [67]. Ethnicity was another extensively studied factor [67, 68, 69, 89], with White patients suggested to be more active [67, 89], ask more questions [69, 89] and have more interactions with physicians [68, 69] than non‐White patients. Educational level also played a role [19, 25, 58, 67, 68, 69, 81, 88]: Patients with higher educational levels tend to be more active, ask more questions and engage more with physicians [19, 25, 58, 67, 69, 75]. Conversely, patients with lower educational levels are less active and express fewer emotions [69]. However, they ask more questions about their insurance or medical costs [68]. Physicians also tend to ask patients with lower educational levels more questions, although interactions with them are shorter compared to those with more educated patients [88]. Socioeconomic status was another important factor, with patients with lower income being less active than those with greater financial resources [68, 69]. Finally, marital status was found to play a role in interactions, with single patients being more active than their married or partnered counterparts [68].

#### 3.5.2 Patients' Health Status and Consultation‐Related Factors

Patients' physical and psychological health can influence their participation and communication in consultations, as reported in seven studies [19, 56, 58, 61, 66, 73, 81]. For instance, anxious or fearful patients tend to show more signs of concern and ask more questions [19, 56, 66, 73, 81]. Additionally, the more symptoms patients are experiencing (e.g., pain and side effects), the more likely they are to focus the conversation on and ask more questions about these symptoms [58, 85]. However, Whisenant et al. [61] found that even if patients report some symptoms before the consultation, they do not always discuss them during their meeting with the physician.

Several consultation‐related factors can also influence patient participation [13, 25, 46, 57, 65, 66, 68, 73, 85]. Longer conversations tend to result in patients expressing themselves more and asking more questions [14, 25, 68, 85]. Patient involvement also varies with topics discussed and cancer stage [13, 46, 57, 65, 66]. Ishikawa et al. [65] found that patients participate more when discussing their test results, Shellenberger et al. [66] observed greater patient participation during case discussions, and Roter et al. [57] noted increased involvement when discussing complementary and alternative medicine. Participation was also found to depend on the timing of the consultation. That is, during initial consultations, patients are usually less involved than during subsequent consultations [13]. This may be because initial consultations are designed to provide patients with medical information, whereas subsequent consultations are more flexible, allowing patients to express their difficulties and side effects [13].

#### 3.5.3 Physician's Influence on Interactions

Twenty‐four studies explored the influence that physicians can have on patients' interactions and behaviours during consultations [10, 13, 19, 25, 57, 61, 62, 63, 64, 65, 69, 73, 74, 76, 78, 80, 81, 82, 83, 87, 88, 89, 90, 91]. In the literature, the patient–physician relationship has often been described as interdependent since both physicians and patients influence each other during their exchanges [76, 90, 91]. For instance, Tang et al. [76] identified eight interaction patterns, including both proactive and passive patterns, during which patients and professionals influence each other (i.e., collaborative, explanatory, agentic, checklist, cross‐purpose, empathic, admonishing and diverging interactions). Patients also express themselves more when physicians show empathy, interest and responsiveness to their words and questions, and a trusting relationship encourages joint decisions. However, the patient has to perceive this open and empathetic attitude as genuine [65]. Hence, patients communicate more when physicians ask more questions and encourage dialogue during consultations [13, 25, 88]. Overall, patients are more active and involved when physicians use PCC methods for communication [10, 74]. Although most included studies indicated a good agreement between physicians and patients [76, 78, 81, 82, 87], some physicians' behaviours sometimes discourage patients from speaking [13, 14, 25, 57, 61, 62, 63, 65, 69, 73, 76, 80, 81, 82, 87, 90, 91]. For example, patients inhibit their communication when they are consulting with physicians who dominate conversations, talk more than them, ask too many questions [13, 25, 61, 65, 69, 76, 80, 82, 83, 91], or appear to be too busy to support patients [73, 76]. Other physician‐related factors that inhibit patients' communication are awkward reactions and a cold attitude [14, 61, 62, 76, 81, 82], as well as discomfort with some topics, particularly psychosocial ones [63, 73]. Patients also express themselves less with physicians who do not acknowledge or perceive their difficulties [64, 90]. Furthermore, highly structured consultations, leaving little space for discussion, tend to constrain patients and prevent them from expressing themselves on other topics [73, 76, 80, 87].

#### 3.5.4 The Influence of Relatives on Patient Behaviour

Among the included studies, 14 addressed the presence of relatives, their role, and the influence they can have during consultations. The influence of the presence of family members on patients' communication was not consistently observed, though. According to Eggly et al. [25], the presence of family members negatively influences patient participation, but only if a close relationship develops between the family member and physician, making the patient feel excluded from the conversation. Contrastingly, this was not observed by Amundsen et al. [19], who indicated that the presence of family members has no influence on patient participation and involvement. However, Street and Gordon [54] showed that accompanied patients are more active than non‐accompanied patients. Nonetheless, family members can act as facilitators for the patient [55, 65, 83], with patients getting family members to raise certain topics [83], provide more details about the information they give [24], or address some emotions [65]. Nevertheless, Buizza et al. [55] noted that patients who are accompanied often experience more distress than those who attend consultations alone.

The articles also show that relatives can get involved in consultations themselves. During consultations, family members also ask questions, usually about the same topics as the patients [13, 19, 24, 25, 54], allowing them to initiate conversations themselves [24, 57, 78, 84] and share their own experiences with the patient's illness [63].

#### 3.5.5 Tools to Enhance Active Patient Communication

Eight studies evaluated the effectiveness of interventions targeting patients' communication behaviours [55, 56, 58, 59, 60, 79, 83, 86], including through the use of a prompt list [55, 86], question booklets [60], discussions with a coach [58, 60, 79], encouragement to speak [60], educational sessions [58, 79], patient‐reported outcomes (PROs) [56, 59, 83] and training for HCPs [47].

A question booklet, combined with encouragement [60], seems to increase patient participation, in contrast to a prompt list [55], which can confine patients to predefined questions that may not fulfil their needs. Hence, encouraging patients to prepare their own questions seems more effective than giving them a predefined list [86]. Working on questions with a coach [60], despite being paired with encouragement and a question list, also does not appear to be effective. This may be because patients find it simpler to work through a question list with a family member rather than a professional [60]. Nevertheless, a coach and an educational intervention can improve active participation [58, 79], leading to more self‐assertion and the greater expression of concerns, symptomsand free‐form questions. In the study by Street Jr. et al. [58, 79], patients asked questions related to the topics covered during the intervention rather than more general questions. PROs have also proven to be effective [56, 59, 83] since they allow patients to discuss more symptoms, engage in longer conversations and ask more questions [56, 59]. However, Heyn et al. [56] noted that physicians more frequently use PROs to explore all aspects of the patient's illness, while Takeuchi et al. [59] observed that physicians have not fully embraced the tool, particularly for addressing psychosocial aspects and patient functioning, potentially due to reluctance or barriers to addressing these domains. Moreover, even when patients report symptoms through PROs, they may not mention them or may even deny them during consultations [83]. Finally, the study by Timmermans et al. [47] aimed to train physicians in the use of PCC. After the training, physicians were found to ask more open‐ended questions, focus more on psychosocial aspects, encourage patients to express themselves and provide more verbal support. Such behaviours positively affect patients, who then participate more actively in consultations, ask more questions and express their opinions, concerns and psychosocial situations more freely. Beyond communication patterns and influencing factors, patients also discussed their perceptions of these interactions and reported varying levels of satisfaction with consultations. This final theme emphasizes how patients evaluate their participation in and the outcomes of these exchanges.

### 3.6 Theme 3: Patient Perceptions of and Satisfaction With Consultations

Although not part of the initial objectives, a third theme emerged from the analysis, highlighting patients' satisfaction and the perceived benefits of active participation during oncology consultations. Specifically, eight studies emphasized the benefits of active patient participation [46, 62, 66, 67, 68, 79, 88, 90], and eight addressed patients' satisfaction with and perspectives of their involvement during consultations [13, 15, 19, 57, 62, 67, 81, 82]. Given the recurrence and relevance of these findings, this additional theme was deemed pertinent to report.

#### 3.6.1 The Benefits of Active Participation

Active participation allows patients to obtain more information and thus gain a better understanding of their cancer [46, 66, 67, 68]. They can then become more involved, make informed decisions [88, 90] and experience fewer decision regrets [88]. Additionally, active communication enables patients to express their preferences, concerns and needs [62, 68, 79], ensuring that these are considered by HCPs on both medical and psychosocial levels [79].

#### 3.6.2 Patient Satisfaction

Most patients report satisfaction with communication and participation in consultations [13, 19, 81, 82, 86], even if they behave passively [13, 82]. Several factors are linked with higher satisfaction [15, 57, 62, 67, 82, 86]: Patients are more satisfied with their interactions when consultations are longer [82] and when the physician asks open‐ended questions [62], is not directive [62] and adopts an empathetic tone [15]. This latter point contrasts with the findings of Siminoff et al. [67] and Ishikawa et al. [62], who found patients less satisfied when physicians display emotional behaviours. Moreover, patients were found to be more satisfied when they receive a high level of information [15], specific topics are discussed [57], and they have ample opportunities to express themselves [67]. Conversely, several factors predict dissatisfaction [15, 62, 67, 81]. Three studies showed that patients who ask many questions are more dissatisfied than others [15, 62, 67], which may be explained by the fact that if patients ask questions, they may feel that the physician has not sufficiently explained themselves or addressed their inquiries [15, 62]. Surprisingly, the presence of psychosocial discussions was not found to be associated with patient satisfaction [62, 82].

## 4 Discussion

In this systematic review, we explored patients' communication behaviours in oncology consultations with healthcare providers, which factors influence communication, as well as patients' perceptions of and satisfaction with patient–physician communication. Our results show that numerous studies have explored these topics. Among the 47 studies included, 14 involved both patients and relatives and investigated their perspectives on patient–physician communication. None of our included studies discussed the communication dynamics between relatives and HCPs, despite evidence in the broader literature suggesting that relatives can influence patient–HCP interactions and dynamics [92]. Our results confirm that patient–physician communication is a dynamic process that can be influenced by several factors, so we must ascertain and fulfil patients' needs while considering how these needs may evolve over time. However, these factors have usually been studied independently, so a global and comprehensive overview is lacking.

The oldest study included was published in 2000 [67], but the foundations of patient–physician communication were laid in the 1950s with Balint's [93] work, emphasizing mutual comprehension and therapeutic relationships. Then, in the 1980s, the position given to patients and patient–physician dynamics evolved, as elaborated in the patient‐centred [3, 5] and shared decision‐making [94, 95] approaches. These two approaches emphasize the importance of directing medical interactions towards patients' needs, values and preferences. Furthermore, these models advocate for an empathetic and collaborative approach, where patients are encouraged to actively participate in their care and decision‐making. As identified in our results, the use of these approaches encourages patients to be more active during consultations and more involved in their care [10, 74].

In our systematic review, two out of three themes identified in the studies can be linked to the patient‐centred and shared decision‐making approaches, and even to the patient‐as‐partner approach: Theme 1 ‘Patient's communication patterns and the topics discussed during oncology consultations’ and Theme 2 ‘Factors influencing patient communication’. The literature involving clinical contexts other than oncology has confirmed the results found for these two themes [96, 97]. However, some factors identified in patient–physician communication models are not present in our results. In Street et al.'s [98] framework, for instance, which describes pathways linking patient–physician communication to health outcomes, communication impacts health both directly and indirectly, primarily through proximal outcomes. These immediate effects can include satisfaction with care, agreement with the doctor, trust and feeling known and cared for. Proximal outcomes mediate the relationship between communication and both intermediate (e.g., access to care, social support and emotional management) and health (e.g., survival, pain control and emotional well‐being) outcomes.

Intermediate outcomes were also not found, except for the role of relatives. Furthermore, physicians' technical or communication skills were not discussed, except for empathic communication. Specifically, medical empathy includes showing empathic concern (i.e., establishing a good relationship, showing care and compassion), favouring patient empowerment (i.e., explaining things honestly and clearly, helping the patient take control, formulating a plan of action with the patient) and reassuring the patient [99, 100, 101, 102]. These aspects were mentioned by the patients in the studies included in our systematic review, underlining the importance of physicians' attitude and relationship in patient–physician communication.

Timing is a central theme in our results. That is, to meet patients' needs, communication must be adapted to the stage of the individual's journey [3, 5]. For example, studies have shown that during the diagnostic period, patients primarily express medical needs, such as the need for information and support. Later, psychosocial needs become increasingly present and expressed, requiring the clinician to adapt accordingly. The level of information and attitude of patients and relatives thus evolves over time [103]. This is perhaps the most challenging factor to consider when adopting a patient‐centred approach.

Wollersheim et al. [87] described two types of HCP communication behaviours: providing space and reducing space, as used by Piccolo et al. [104] when analyzing follow‐up visits. Providing space involves responses that allow patients to elaborate on their cue or concern, while reducing space involves responses that limit the patient's opportunities to further discuss their cue or concern. Even if current patient–physician communication models and theories promote the use of strategies to provide space [3, 5, 94, 95], both providing and reducing space are reported in our results, and both can have a positive or negative impact on the patient depending on their characteristics. This impact can also be related to several factors, including time constraints, systemic and structural factors (e.g., healthcare systems and policies might prioritize efficiency and productivity over PCC), medical training, culture and communication skills [70, 105].

Additionally, several studies emphasized patients' satisfaction with and the perceived benefits of active participation during oncology consultations. While greater involvement is often linked to improved information exchange and empowerment—contributing to patient satisfaction—many patients reported being satisfied, even when adopting a more passive role [106, 107]. These findings suggest that satisfaction is highly individual and is shaped by factors such as cultural background, personal preferences and sociodemographic characteristics. This underscores the need to tailor communication strategies to each patient's expectations and interaction style.

Finally, cultural and contextual healthcare factors may also play a critical role in shaping patient–physician communication. This point was not identified in the present systematic review but warrants discussion. Specifically, studies conducted in different countries have suggested that cultural norms influence patients' preferred level of participation, the expression of emotions and expectations for the physician's role [108]. For instance, in some cultures, patients may adopt a more deferential attitude towards medical authorities and prefer a physician‐led decision‐making process, whereas in others, open dialogue and shared decision‐making carry higher value. Similarly, healthcare system characteristics—such as consultation duration, the physician's workload and continuity of care—vary worldwide and can either facilitate or constrain PCC and satisfaction [109, 110]. These findings underscore the need to interpret communication behaviours within their sociocultural and systemic context rather than applying a uniform model across settings.

### 4.1 Study Limitations

Our study had several limitations that should be discussed. First, most of the included studies in this review were conducted in the United States or other Western countries, which may have limited the generalizability of the results to other healthcare systems and cultural contexts. Healthcare systems and policies, physician practices and communication styles differ across nations [111], necessitating exploration in diverse settings and countries. These differences can influence how medical information is communicated and received, as well as patients' expectations regarding clinical interactions [112]. For instance, in some contexts, patients may take a more passive role in their care or prefer collective rather than individual interactions. Similarly, constraints related to time, resources, or professional training can affect the quality and frequency of communication. Therefore, strategies considered effective in Western contexts may require adaptation to be relevant and acceptable in different healthcare systems and cultural settings.

Second, regarding participants' characteristics, the chosen studies only included patients without speech disorders and most of the studies (40 out of 47) included solely doctors as HCPs. This focus on physicians provides valuable insights into their perspectives and practices, but it results in the experiences and viewpoints of other HCPs, who also play critical roles in patient care, being overlooked.

Third, the heterogeneity in study designs, variables, outcomes and reporting processes, along with the inclusion of qualitative and mixed‐methods studies, precluded a formal meta‐analysis and a synthesis of effect sizes across studies. Across the included studies, those following qualitative and mixed‐methods designs were generally of high methodological quality, warranting confidence in their results. However, RCTs and quantitative descriptive studies displayed more methodological variability, including limitations in randomization, blinding, sample representativeness and intervention implementation, warranting a more cautious interpretation of their results.

### 4.2 Theoretical and Research Perspectives

**Figure 2:**
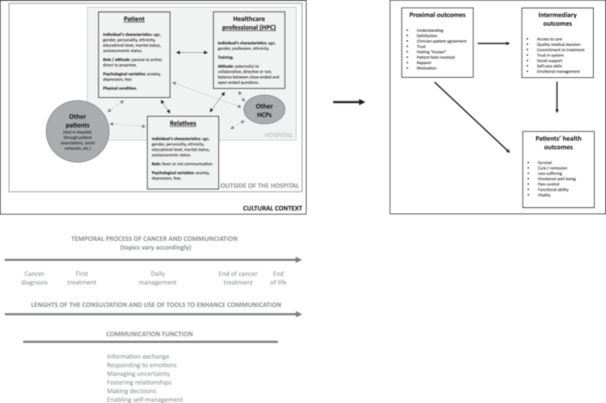
Comprehensive and graphical summary of healthcare communication.

In this systematic review, we presented an overview of the factors impacting patient–physician communication. Communication is one of the most studied subjects in oncology since it can be used to improve patient care. As discussed, several models of care exist, including the patient‐centred [3, 5] and shared decision‐making [94, 95] approaches. Additionally, there are models of patient–physician communication that underline the importance of empathy [98, 99, 100, 101, 102]. In Figure 2, we present a summary of the factors identified in our review and presented in the cited models, especially those discussed in the work of Street et al. [98]. Patient–physician communication can be influenced by several factors: individuals' characteristics (e.g., age and gender), role or attitude during the consultation (passive or active vs. paternalistic or collaborative), HCPs' training, patients' physical condition and patients' psychological variables (e.g., anxiety, depression and fear). Relatives, other HCPs, and other patients can also influence patient–physician communication [94, 113, 114], as can the cultural context in which the communication occurs [113, 114]. Relatives' individual characteristics and psychological health can also influence communication, as well as their behaviours during the consultation (e.g., asking many questions, being neutral or passive), which may improve or harm communication. As Street et al. [98] described, and as partially observed in our systematic review, we believe that all these variables of patient–physician communication can impact patients' health outcomes (i.e., their survival, decreased suffering and functional ability) through intermediary outcomes (i.e., access to care, the perceived quality of medical decisions, commitment to treatment, trust in the system, social support, self‐care skills and emotional management) and proximal outcomes (i.e., understanding, satisfaction with care, clinician–patient agreement, trust, feeling understood, feeling involved, the relationship and motivation). In addition, timing, the duration of communication, the use of tools (e.g., booklets, prompt lists and coaching) and the function of communication (i.e., informational exchange, decision‐making, uncertainty management, relationship fostering and self‐management encouragement) significantly impact patient–HCP–relative communication.

Compared to other clinical models, the graphical summary presented in Figure 2 presents a global understanding of which of an individual's characteristics, variables and attitudes influence their health outcomes through health communication.

In future research, the graphical summary presented in Figure 2 should be tested to improve our understanding of the complex relationships among the different variables and persons involved in patient–physician communication and patient satisfaction. The summary should also be tested in various cultural contexts and healthcare systems to identify potential needed adaptations. To comprehensively assess the impact of patient–physician communication on patient satisfaction, a longitudinal study can be conducted using both self‐reported and observational measures. The impact of physicians' characteristics and outcomes should also be assessed carefully. However, to conduct such a study, we believe that the research team should include the patient as a partner in the research.

### 4.3 Practical Implications

The findings of this review, contextualized within scientific literature and summarized in Figure 2, have several practical implications for HCPs, representing basic recommendations that may not be implemented universally as yet:

A. Tailoring communication to patients' individual characteristics and psychosocial needs (as shown in Figure 2) may enhance their satisfaction, engagement and understanding.
  For instance, at the beginning of a consultation, clinicians could explicitly assess a patient's preferred level of information and emotional support explicitly by, for example, asking whether the patient wishes to receive detailed prognostic information or rather focus on immediate treatment options. Regarding emotional aspects, HCPs could be trained to notice emotional cues (e.g., expressions of anxiety or fear, nonverbal behaviours such as facial expressions or posture) and respond appropriately by, for example, incorporating empathy into their communication, and pausing to validate concerns before continuing, which can help enhance trust and engagement [115, 116]. Involving relatives or caregivers in consultations, when appropriate, could aid in addressing the contextual and social factors mentioned in Figure 2 and enhance comprehension, support shared decision‐making and improve adherence to care plans.
  In practice, HCPs could invite a patient's spouse or adult child to attend key appointments, with the patient's consent and without constraining the relative, to help clarify treatment options and ensure a shared understanding of care plans. Relatives could be encouraged to take notes or ask questions during consultations so that they can facilitate adherence to complex medication regimens at home. This collaborative approach may also help in identifying psychosocial barriers, such as familial stress or logistical challenges, that might otherwise remain unaddressed [117]. It could also empower the relatives, who may sometimes feel both helpless and burdened at various points in the care pathway. Additionally, this would also contribute to recognizing the role of the caregiver and facilitate the patient‐relative communication.
B. Integrating tools such as prompt lists, coaching, or informational booklets could help address specific needs (e.g., patient anxiety, uncertainty or low health literacy), providing concrete support to improve communication outcomes.
  Clinicians could provide patients with a structured ‘question prompt list’ before the consultation to help them better articulate their main concerns and information needs. In settings where anxiety or uncertainty is prevalent, patients might also benefit from brief communication coaching sessions focusing on how to express priorities and ask clarifying questions. Similarly, distributing tailored informational booklets that define medical terminology and outline expected treatment steps can empower patients with low health literacy by improving their understanding and recall of the information discussed during appointments. At the end of the communication, in addition to giving patients a hard‐copy booklet, the physician could also summarize the key points of the consultation and ensure that the patient and/or their relative has understood them [118].
  By linking these actions directly to the factors identified in our review, HCPs can move beyond general patient‐centred care principles towards targeted, evidence‐informed strategies, which could result in optimized patient–provider communication and the ultimate improvement of health outcomes. To aid in this endeavour, HCPs could also benefit from targeted training in communication skills, including medical empathy, which could help them consider patient characteristics, diverse needs and psychosocial contexts more effectively, as shown in the model described in Figure 2.

## 5 Conclusion

To our knowledge, no study has summarized how patients communicate and behave with HCPs and which factors influence this communication. Hence, we conducted this systematic review to improve our understanding of patient–physician dynamics. More research involving patients, relatives and different HCPs is needed. We strongly encourage a comprehensive approach to studying the communication factors to tailor patient‐centred approach that meets the needs of the patient.

## Author Contributions

**Pauline Justin:** conceptualization, data curation, formal analysis, investigation, methodology, resources, software, validation, visualization, writing ‐ original draft, writing ‐ review and editing. **Valentyn Fournier:** conceptualization, data curation, investigation, methodology, resources, software, validation, writing ‐ review and editing. **Ambre Naeyaert:** data curation, investigation, methodology, resources, software, validation, writing ‐ review and editing. **Lisa Laroussi‐Libeault:** writing ‐ review and editing. **Christelle Duprez:** review and editing. **Pascal Antoine:** writing ‐ review and editing. **Kristopher Lamore:** conceptualization, methodology, project administration, resources, software, validation, visualization, writing ‐ original draft, writing ‐ review and editing.

## Ethics Statement

The authors have nothing to report.

## Conflicts of Interest

The authors declare no conflicts of interest.

## Supporting information

Supporting Information 1.

Supporting Information 2.

## Data Availability

Data sharing is not applicable to this article as no new data were created or analyzed in this study.
