# Supplementary material for: Exploring Communication Dynamics Between Patients and Healthcare Providers in Oncology: A Systematic Review
Source: Health Expect. 2025 Dec 11;28(6):e70519. doi: 10.1111/hex.70519 (PMC12696885; doi:10.1111/hex.70519)
Supplement: Supplementary file 1 — Supporting Information 1. [file HEX-28-e70519-s001.docx]

**Supplementary material 1**. Characteristics of the included studies.

| **Ref.** | **Author, year of publication, country** | **Funding** | **Research design** | **Research design (according to the Mixed-Methods Appraisal Tool) and methods** | **Study purpose** | **Sample characteristics** | **Measures** | **Results*** |
| --- | --- | --- | --- | --- | --- | --- | --- | --- |
| [70] | Alexander et al., 2012, USA | National Cancer Institute | Observational study | Mixed methods, with audio-recorded consultations | To describe and quantify the content of a consultation in terms of the exchange of information, as well as identify the patient and provider characteristics associated with the elements under discussion | **Participants**: Patients and hematologists **Sample size**: *N* patients = 236; *N* hematologists = 40  **Gender**: 53% male (patients); 34% male (hematologists) **Age range**: Patients: *M* = 55, range: 20–79 years; hematologists: *M* = 47, range: 30–70 years  **Cancer localization:** Hematologic malignancy (lymphoma, leukemia, myelodysplastic syndrome, multiple myeloma, other) **Cancer grade**: N/A **Treatment**: N/A **Presence of a caregiver**: Yes, but not specified | Sociodemographic data Consultation duration  Number of medically related questions asked by patients and relatives who accompanied patients to their visits A specifically developed communication evaluation tool to measure the components of patient–physician verbal interactions during consultations | Patients and their support people asked a median of 23 questions during the consultations, with a range of 0–78 questions.  The treatment’s impact on the patient’s quality of life was more likely to be discussed if the patient and support person asked more questions than average. Quantitative prognostic discussions of mortality without hedging were associated with lower patient education and a higher than average number of questions asked by the patient or support person but were most clearly associated with which physician was being seen. Quantitative prognostic discussions of a cure without hedging were associated with the non-White patient race, lower education, and a higher number of questions asked. |
| [89] | Amelung et al., 2020, England | Cancer Research UK | Qualitative study | Qualitative, with video-recorded consultations | To understand doctor–patient communication about the importance of persistent or new problems and their potential impact on primary care | **Participants**: Patients and physicians **Sample size***:* *N* patients = 20; *N* physicians = 7 **Gender**: 44% male (patients); 76.4% male (physicians) **Age range**: Patients: *M* = 66.5, range: 50–96 years; physicians: *M* = 48.2, range: 32–60 years **Cancer localization**: N/A **Cancer grade**: Primary care **Treatment**: N/A **Presence of a caregiver**: No | Thematic analysis approach to identify any new or persistent problems relevant to the cancer, as reported by the patients | Doctors and patients defined and expressed significance differently—the doctor expressed it medically, while the patient expressed it personally. Potentially detrimental patient behaviors and experiences occurred when the patient’s expressed significance of a presenting problem was misaligned with the doctor’s expressed significance, and this was not resolved during the conversation.  Overall, 25% of consultations involved a misalignment that was easier to resolve when it was a difference in knowledge but did not involve emotional factors. |
| [19] | Amundsen et al., 2018, Norway | Northern Norway Regional Health Authority; Helse Nord RHF | Observational and quantitative study | Mixed methods, with audio-recorded consultations and questionnaires | To explore how Norwegian cancer patients actively participate in consultations by asking questions and expressing emotional signals or concerns, as well as to what extent this behavior is associated with shared medical decision-making | **Participants**: Patients and physicians  **Sample size***:* *N* patients = 31  **Gender**: 36% male  **Age range**: *M* = 57 (*SD* = 14), range: 18–75 years  **Cancer localization**: Colon or anus, breast, lung, testis, other, missing data  **Cancer grade**: N/A  **Treatment**: N/A  **Presence of a caregiver:** Yes (*N* = 7) | Sociodemographic data  Questions from patients or caregivers  Emotional cues (coded according to VR-CoDES)  Concerns expressed by the patients | The number of questions asked by the patients varied from one to 63 questions (mean = 17, *SD =* 15, median = 11).  Caregivers were present in seven of the consultations and played an active role by asking questions during five consultations.  Caregivers raised fewer questions than patients.  When including caregiver questions, the mean number of questions per consultation was 20.  The most frequent types of questions were treatment (261 questions, 42%) and practical issue (154 questions, 24%) questions.  Each consultation had 8.7 questions (*SD =* 10.4) about treatment and 5.0 questions (*SD =* 5.3) about practical issues.  Seven of the 615 questions (1%) referred to prognoses, occurring in four of the 31 consultations.  Four of the 615 questions (0.7%) dealt with treatment options, occurring in two of the 31 consultations.  Pre-consultation anxiety was significantly associated with the number of questions asked.  Educational level was significantly related to the number of questions asked, but only when including caregiver questions.  Patients asking more questions expressed significantly more cues and concerns than those asking fewer questions (*r*[29] = .47, *p* = .007).  Pre-consultation anxiety was the only factor significantly associated with the number of cues and concerns expressed in both the univariate and multivariable regression models. |
| [69] | Beach and Dozier, 2015, USA | National Institutes of Health; National Cancer Institute | Observational and quantitative study | Mixed methods, with video-recorded consultations and questionnaires | To examine how patients raise their concerns, how physicians respond to patient-related actions, and the implications of these factors for communication satisfaction | **Participants**: Patients and physicians  **Sample size**: *N* patients = 44; *N* physicians = 14  **Gender**: 45.5% male (patients); 66.7% male (physicians)  **Age range**: Patients: *M* = 58.6, range: 23–86 years; physicians: *M* = 45.5, range: 35–57 years  **Cancer localization**: N/A  **Cancer grade**: N/A  **Treatment**: N/A  **Presence of a caregiver**: N/A | Verbal actions initiated by patients (i.e., lexical references to fear, uncertainty, or hope; non-lexical indirect actions, e.g., stating a concern, seeking clarification, announcing good news) | The authors identified an average of 24.31 patient-initiated actions per interview (approximately 10 uncertainty, nine fear, and six hope).  Thirty-nine of the 44 interviews included at least two or more patient-initiated actions.  Most patient-initiated actions (87.5%) were indirect.  Direct and indirect patent-initiated actions related to uncertainty were most frequent (39.6%), followed by direct and indirect fear (36.1%) and direct and indirect hope (22.5%).  Patients with higher educational levels displayed significantly more patient-initiated actions than patients with lower educational levels.  The number of patient-initiated actions was not related to income, gender, ethnicity, age, or marital status.  No significant differences were found between the types of cancer and frequency or the types of patient-initiated actions.  Most patient-initiated actions relating to fear occurred before the physical exam (76.7%).  Patients with higher scores on the pre-visit fear index were more likely to initiate direct fearful actions during the interview and to ask more questions.  Most fears about cancer were expressed indirectly.  Most patient-initiated actions relating to uncertainty occurred before the physical exam (54.5%).  Patients with higher scores on the pre-visit uncertainty index were no more likely to initiate actions about uncertainty than patients with lower scores.  Overall, 82.7% of patients’ uncertainty actions were indirect.  Direct uncertainty references (e.g., ‘‘I’m not certain,’’ ‘‘I don’t know’’) were more frequent (17.2%) than direct fear (8.0%) or hope (11.5%) references.  Patients used questions to discuss their uncertainty (e.g., seeking more information or clarification) more frequently (14.8%) than to address fear (11%) and hope (3%).  The post-visit uncertainty index revealed that patients who initiated more actions relating to uncertainty during the interview were significantly more uncertain after the medical interview.  Most patient-initiated actions relating to hope occurred before the physical exam (72.5%).  Patients with higher scores on the pre-visit hope index initiated more hopeful actions during the interview. Such hopeful actions were generally indirect (5%) rather than explicit (e.g., hope, hopeful, hopefully).  Patients expressed minimal or no emotions 98.2% of the time when initiating social actions. |
| [81] | Bottacini et al., 2017, Italy | N/A | Randomized controlled trial, qualitative and quantitative study | Mixed methods, with audio-recorded consultations and questionnaires | To assess whether there was a difference in the efficacy of a Question Prompt Sheet (QPS) versus the Question Listing (QL) method in increasing the number of questions that breast cancer patients in the early stages of the disease ask during their initial encounter with an oncologist. | **Participants**: Patients and oncologists  **Sample size**: *n* patients = 308; *n* oncologists = 20  **Gender**: 100% female (patients); 85% female (physicians) **Age range**: Patients: *M* = 55.6 years, (*SD* = 16.9); physicians: N/A  **Cancer localization**: breast  **Cancer grade**: 0–III  **Treatment**: N/A  **Presence of a caregiver**: Yes (72%) | Sociodemographic data  Consultation duration and number of questions  Satisfaction questionnaire, anxiety | The duration of consultations ranged between 12 and 129 min, with an average of 49 min.  (1) A patient enrolled in the QL group asked, on average, 1.7 more questions than a patient enrolled in the QPS group. (2) The presence of a companion reduced the number of questions that patients asked (about 5). (3) Despite their assigned group, on average, patients asked more questions during longer consultations (a new question every 3 min).  Patients commonly asked about illness management (41.2%) and administrative issues (32.5%). There were no significant differences between the two groups.  Patients in the QPS group were significantly less satisfied with the information they received compared with those in the QL group. |
| [54] | Buizza et al., 2021, Italy | N/A | Randomized controlled trial, quantitative study | Randomized controlled trial, with audio-recorded consultations and questionnaires | To evaluate whether the introduction of a communication tool, with or without support, impacts the number of questions asked by patients during consultations and subsequent psychological and relational outcomes | **Participants**: Patients and oncologists  **Sample size**: *N* patients = 308  **Gender**: 100% female (patients)  **Age range**: *M* = 56.3 (*SD* = 10.3), range: 18–75 years  **Cancer localization**: Breast  **Cancer grade**: Stage I, stage II, stage III, missing  **Treatment**: Conservation surgery, mastectomy, no breast reconstruction  **Presence of a caregiver**: Yes, but not specified | Sociodemographic data  The number of questions asked by patients during the consultation  The presence or absence of a companion | Patients under the Question Listing condition asked more questions than those in the Question Prompt List group for all topics excluding “prevention.”  Consultation duration was the same for all groups, and no difference was found for the interaction or clinical variables.  In the Question Prompt List group, unaccompanied patients asked more questions than accompanied patients, especially about “treatment” and “bureaucracy.”  In the Question Listing group, unaccompanied patients asked, on average, more questions than accompanied patients about all topics excluding “prognosis” and showed greater involvement during the consultation.  Accompanied patients had significantly higher scores than unaccompanied patients on clinical symptomatology scales.  Unaccompanied patients asked more questions than accompanied patients about all topics apart from “prognosis” and showed greater involvement during the consultation.  Unaccompanied patients had significantly lower scores than accompanied patients on clinical symptomatology scales.  Accompanied patients in the Question Listing group asked more questions than their counterparts in the Question Prompt List group and showed more interest in “prognosis” topics than accompanied patients in the Question Prompt List group.  The most frequent topic of discussion in both groups was “treatment.” |
| [14] | Butow et al., 2002, Australia | Australia National Health; Medical Research Council | Observational and quantitative study | Quantitative, with audio-recorded consultations and questionnaires | To explore the frequency of direct and indirect informational and emotional cues that occur during a cancer consultation, the patient characteristics that predict cue emission, and the impact of the physician's response on subsequent cue emission, patient satisfaction, and anxiety | **Participants**: Patients and doctors  **Sample size***:* *N* patients = 298  **Gender**: 57.1% male  **Age range**: *M* = 56.18, range: 18–83 years  **Cancer localization**: Genitourinary, breast, gastrointestinal, lymphoproliferative, skin, other  **Cancer grade**: N/A  **Treatment**: N/A  **Presence of a caregiver**: N/A | Sociodemographic data  Patient behaviors directly and indirectly indicating a need for information or emotional support | Patients asked a median number of 11.45 questions and a mean number of nine questions.  The most frequent topics were history and symptoms, diagnosis, treatment, prognosis, or other medical issues.  The median number of cues per consultation was two cues, and the mean was 3.38 cues.  Most cues were related to treatment issues.  Patients gave informational cues almost twice as often as they gave emotional cues.  Consultation duration ranged from 4.83 to 44.01 min, with a mean duration of 19.06 min.  There was no relationship between the consultation duration and the number of emotional cues given.  The longer the consultation, the more informational cues were given and the more questions were asked.  Patients who asked more questions also tended to give more indirect cues for information.  The duration of the consultation was significantly associated with the number of informational cues given, while patient age and gender were significantly associated with the number of emotional cues given.  Younger and female patients gave more cues. |
| [83] | Consolandi et al., 2024, Italy | None | Qualitative and quantitative study | Quantitative, with audio-recorded consultations and questionnaires | To collect quantitative data on the level of engagement of pancreatic adenocarcinoma patients; to study the characteristics of communication between physicians and pancreatic ductal adenocarcinoma patients and the rate of patients' understanding of the information received from physicians; to investigate the associations between patients and disease-related variables, communication, and understanding, as well as the level of engagement and compliance of patients | **Participants**: Patients and oncologists **Sample size***:* *N* patients = 30 **Gender**: 50% female **Age range**: *M* = 63.7, range: 60–67 years **Cancer localization**: Pancreas  **Cancer grade**: Resectable, borderline resectable, locally advanced, metastatic **Treatment**: Chemotherapy, surgical resection **Presence of a caregiver**: Yes | Thematic analysis of the interaction | The mean time of the visits was 31.1 min, with a significantly longer duration for oncology visits than for gastroenterology or surgery visits. There were 6,780 conversational turns, 50.6% doctors, 37.4% patients, and 12% caregivers.  The difference in the time of participation in the conversation was more relevant when examining the percentage of time spent speaking during the visit, with a median of 77% of the total time for doctors, 13% for patients, and 2% for caregivers. The median percentage of conversations held by the patients was 14% for female patients and 9.5% for male patients. Female caregivers intervened more often and for longer during doctor–patient interviews than their male counterparts. |
| [75] | D'Agostino et al., 2020, USA | National Cancer Institute of the National Institutes of Health; The Duncan Family Institute for Cancer Prevention; Risk Assessment at MD Anderson | Observational and quantitative study | Mixed methods, with audio-recorded consultations and questionnaires | To examine discussions about mastectomy during natural interactions between women with sporadic breast cancer and their oncology healthcare providers when making treatment decisions | **Participants**: Patients and providers  **Sample size***:* *N* patients = 28  **Gender**: 100% female  **Age range**: *M* = 53.8, range: 28–70 years  **Cancer localization**: Breast  **Cancer grade**: Stage 0 to III unilateral breast cancer  **Treatment**: N/A  **Presence of a caregiver**: Yes (*N* = 3) | Sociodemographic data  Patient-related coding categories included who initiated the mastectomy discussion, the context under which the topic of mastectomy emerged, underlying factors that appeared to be driving interest in mastectomy, and patients’ level of engagement during mastectomy discussions | Half of the mastectomy discussions were initiated by the patient *(n* = 13; 46.4%) or the oncology provider *(n* = 12; 42.9%).  A few mastectomy discussions were initiated by a significant other or family member *(n* = 3; 10.7%). Interest in pursuing mastectomy to reduce the risk of future breast cancer was the most frequently observed code (83.3%), either to avoid going through the cancer experience again (22.2%) or for cosmetic or appearance-related reasons (16.7%).  At least one active patient participation behavior code was observed in 71.4% of cases (*n* = 20), patients most frequently asked questions to get additional information or clarify information (*n* = 15; 53.6%), patients used assertive communication in about 35.7% of cases (*n* = 10), and patients explicitly expressed an emotion or concern in about 21.4% of discussions *(n* = 6). |
| [24] | Del Piccolo et al., 2014, Italy | N/A | Observational and quantitative study | Quantitative, with audio-recorded consultations and questionnaires | To provide the first descriptive evidence for the characteristics of unaccompanied and accompanied Italian breast cancer patients attending their first consultation after surgery and to analyze the contributions of accompanying persons to the type and quality of questions asked during the consultation | **Participants**: Patients, oncologists and companions **Sample size***:* *N* patients = 70; *N* companions = 48 **Gender**: 100% female (patients); 58.3% male (companions) **Age range**: N/A **Cancer localization**: Breast **Cancer grade**: N/A **Treatment**: N/A **Presence of a caregiver**: Yes (*N* = 48) | Sociodemographic data The number and type of questions (i.e., symptoms, etiology, prognosis, prevention, treatment, or administration) asked by patients or their relatives.  For companions: the function of questions (i.e., to repeat or verify something already said by the oncologist, to introduce new information requests by changing topic, or to help complete the collection of information). For companions, after listening to each audio-tape: their role, i.e., observer (contributed to the consultation less actively than the patient and played a more passive role), partner (adopted a shared role), and advocate (contributed actively to the consultation, encouraging the patient to ask questions) | Overall, 69% *(n* = 48) of patients attended consultations with one or two relatives. Patients with a companion showed a greater tendency to play a passive role in the decision-making process than those who attended the consultation alone.  More than half of the participants in each group expressed a role preference for shared decision-making.  The number of questions and their distribution over the six content categories were similar in both groups of patients.  Most of the questions focused on illness management (44%) and administrative procedures (37%). Companions asked significantly fewer questions per consultation than their patients but gave priority to the same topics.  Most of the questions asked by companions introduced requests for new information (53%) or helped to complete the obtained information (38%).  Checking the information provided by the oncologist was relatively rare (9%).  In terms of roles, 67% of companions adopted a shared role, only 12% played a passive role, and the remaining 21% were active advocates. |
| [71] | Del Piccolo et al., 2019, Italy | N/A | Observational and quantitative study | Mixed methods, with audio-recorded consultations and questionnaires | To report the number and type of emotional expressions (cues or concerns) raised by breast cancer patients and to identify the impacts of context, patient characteristics, and the patient–physician interaction on emotional expression | **Participants**: Patients and oncologists  **Sample size***:* *N* patients = 308  **Gender**: 100% female  **Age range**: *M* = 57 (*SD* = 10.3)  **Cancer localization:** Breast  **Cancer grade**: Early-stage non-metastatic breast cancer (from stage 0 to III)  **Treatment**: N/A  **Presence of a caregiver**: N/A | Sociodemographic data  The number and type of emotional expressions | Cues were most often expressed as verbal hints to hidden emotions using emphasis, unusual descriptions of symptoms, metaphors, and expressions of uncertainty and hope (59%) or via the use of nonverbal expressions like crying or sighing (12%); neutral expressions alluded to stressful life events and conditions (9%) or a vague expression of emotion (7%).  The remaining expressions were emphasized descriptions of physiological or cognitive correlates of unpleasant emotional states (5%), repetitions of previous cues (4%), or referrals to past concerns (5%).  Concerns often referred to worries about the side-effects of chemotherapy, the course of the illness and its consequences, the fear of disease relapse, or the explicit expression of an emotion.  Patients categorized by the oncologist as anxious expressed more cues or concerns. |
| [25] | Eggly et al., 2006, USA | National Cancer Institute | Observational and qualitative study | Randomized controlled trial, with audio-recorded consultations and questionnaires | To identify the questions asked by patients and their caregivers during stressful oncology encounters in the USA and to analyze the extent to which personal and demographic characteristics and independent assessments of the relationship between oncologist, patient, and carer are related to question asking | **Participants**: Patients and oncologists **Sample size***:* *N* patients = 28 **Gender**: 54% male **Age range**: *M* = 59.6 (*SD* = 11.81) **Cancer localization**: Lung, breast, mesothelioma, pancreatic, biliary, liver, multiple myeloma, colon, throat, rectal **Cancer grade**: N/A  **Treatment**: N/A **Presence of a caregiver**: Yes (*N* = 24) | Sociodemographic data Dyadic interpersonal relationships | Across 28 interactions, patients and their companions asked a total of 705 questions; the number of questions per interaction ranged from three to 65 (*M* = 25,18), with 43% asked by patients and 57% by companions.  An analysis of the 24 interactions in which companions were present showed that companions asked 63% of the total questions. The average number of questions per interaction asked by patients was significantly fewer than the number asked by companions.  Of the 269 treatment-related questions asked during interactions where there was at least one companion, 72% were asked by companions. During the interactions with a single companion, companions asked 68% of the treatment-related questions. Among both patients and companions, treatment was the most frequent question topic (32.2% of patient questions; 48.0% of companion questions), followed by diagnostic testing (15.6% patients vs. 8.4% companions), diagnosis (10.3% patients vs. 6.9% companions), and prognosis (6.0% patients vs. 7.4% companions). Overall, 24% of patients’ most frequent question topics were diagnostic testing, and 12% of companions’ questions were on this topic. Among the treatment questions, the most frequent topic asked by both patients and companions was logistics (46% patients vs. 40% companions), followed by outcomes (25% patients vs. 20% companions) and eligibility or availability for new trials and drugs (10% patients vs. 14% companions). The frequency of questions asked was unrelated to patient or companion gender, race or ethnicity, marital status (married vs. other), relationship with companions (spouse vs. other), or gender concordance or discordance between the patient and companion. The frequency of questions asked by the patients was significantly correlated with age, level of education, and self-reported knowledge of behavioral science. |
| [88] | Eggly et al., 2015, USA | National Cancer Institute | Qualitative and quantitative study | Mixed methods, with video-recorded consultations | To study differences in oncologist–patient communication during clinical trial participant offers in oncology consultations with African American and White patients | **Participants**: Patients and physicians  **Sample size***:* *N* patients = 22  **Gender**: 59% male  **Age range**: *M* = 63.2 (*SD* = 10.4)  **Cancer localization**: Digestive, genital, breast, oral, myeloma, respiratory  **Cancer grade**: N/A  **Treatment**: N/A  **Presence of a caregiver**: N/A | Interaction duration | The mean word count of the entire visit was less for African American patients than White patients.  Risks were mentioned less frequently among African American patients, and the mean word count when risks were mentioned was also less among African American patients.  There were more frequent mentions of voluntary participation among African American patients, and the mean word count for voluntary participation with African American patients was also marginally greater. |
| [57] | Eggly et al., 2017, USA | National Cancer Institute; National Institute of Health | Randomized controlled trial, observational study, and quantitative study | Randomized controlled trial, with audio-recorded consultations and questionnaires | To evaluate an intervention designed to increase active patient participation and other communication-related outcomes in interactions between Black patients and non-Black physicians | **Participants**: Patients and oncologists **Sample size***:* *N* patients = 114; *N* oncologists = 18  **Gender**: 91.2% female (patients); 56% male (oncologists) **Age range**: Patients: *M* = 58.89 (*SD* = 10.35), range: 30–85 years; oncologists: *M* = 46.76 (*SD* = 10.60) **Cancer localization**: Breast, colorectal, lung **Cancer grade**: N/A **Treatment**: N/A **Presence of a caregiver**: N/A | Sociodemographic data Patients’ active participation in seven behaviors: asking the doctor to explain treatments, procedures, and other topics in greater detail; asking the doctor for treatment recommendations; asking the doctor about options for treatment and other topics; informing the doctor about what they liked about the treatment options and other topics; telling the doctor their preferences; freely expressing concerns and worries; expressing opinions The frequency count of three communication behaviors that reflected active participation: asking questions, making assertions, and expressing concerns Oncologist–patient talk time ratio | Patients in the Question Prompt List-Only arm were rated as participating more actively than patients in the usual care arm.  There was no significant difference between patients in the Question Prompt List-plus-Coach arm and those in the usual care arm. Patients in the Question Prompt List-Only arm made more active participation statements than patients in the usual care arm.  There was no significant difference between patients in the Question Prompt List-plus-Coach arm and those in the usual care arm.  Patients in the Question Prompt List-Only arm also made more active participation statements than those in the Question Prompt List-plus-Coach arm.  There were no significant differences in talk time ratios between the intervention arms and the usual care arm. A chi-squared analysis of differences in patient perceptions of their role in treatment decisions across the three study arms was nonsignificant. |
| [62] | Fagerlind et al., 2008,  Sweden | Swedish Cancer Society | Qualitative study | Qualitative, with audio-recorded consultations | To characterize the content of patient–physician communication in standard oncology care | **Participants**: Physicians and patients  **Sample size**: *N* = 19 patients; *N* = 6 physicians  **Gender**: 42% female (patients)  **Age range**: Patients: *M* = 65, range: 40–84 years  **Cancer localization**: Gastrointestinal (pancreatic, anal, colon, rectal)  **Cancer grade**: Adjuvant, curative, or palliative  **Treatment**: Chemotherapy, radiotherapy, or best supportive care  **Presence of a caregiver**: N/A | Qualitative content analysis  Time analysis based on three themes: medical and physical aspects, patient-centered aspects, and other aspects of communication | Patients sometimes stated that they were doing well. However, later, they often described the extensive problems they were facing.  Many patients wanted to know their prognosis and wanted more information about what to expect during the last period of their lives.  Sick leave was introduced both by physicians and patients and was often discussed at the end of the consultation.  Drug prescription often occurred at the end of the consultation and was likewise initiated by the physicians and the patient.  Some patients struggled with insomnia, while others expressed emotions like fear and/or anxiety. Some patients worried about what had caused the tumor, while others were afraid of dying and/or leaving their relatives behind; yet others feared a relapse of the tumor.  Some patients described how their frame of mind was affected by the disease. Some patients were depressed, while others expressed feeling emotionally fatigued and indolent, with deteriorated self-confidence, after being diagnosed with cancer.  Some patients expressed that they tried to live as normally as possible. One patient said that her treatment-related facial rashes affected her relationship with other people, exemplified by how they had stopped her from going to a graduation party. Another patient described how he had experienced friends’ withdrawal.  Some patients expressed how important their work was to them. One patient described how work positively affected her wellbeing, and ways for her to continue working were discussed, although she was very ill.  Quality of life issues were seldomly explicitly addressed: Three consultations included the word “quality of life.”  Some patients described how they had come to accept the disease, while others expressed how they had become used to taking drugs.  The relatives’ coping was sometimes discussed, a topic that was most often physician-initiated. Some patients tried to optimize the relatives’ coping by informing them a little at a time. One patient said that her husband would not talk about her disease and their future. Some relatives attending the consultation said that they could continue their normal lives, while others were more negatively affected and were on sick leave.  Several patients expressed a desire to get psychosocial counseling.  Some patients also expressed the importance of the support they received from relatives, friends, working companions, and neighbors.  Many patients experienced how their families and friends wanted to discuss their own experiences with cancer, which were often completely unrelated to the patients’ current situation.  Many patients wanted their physicians to clarify the accuracy or inaccuracy of information.  Patients’ and relatives’ trust in physicians and health care manifested itself in a few consultations. One patient expressed that he was willing to endure whatever treatment necessary to have a better chance at being permanently cured. Another example was a patient’s wife who strongly expressed her hope that the physician could keep her husband alive for a long time.  Some patients expressed critical comments toward health care. Some were critical of how physicians had communicated with them. Others criticized the way that they had been treated. One male patient strongly criticized the lack of information before radiation therapy, having been informed after the therapy that the treatment could lead to sterility. |
| [82] | Fritz et al., 2025, USA | National Cancer Institute | Qualitative study | Qualitative, with audio-recorded consultations | To characterize surgeon-patient communication about rectal cancer treatment and to describe the current state of communication and identify areas of improvement for shared decision-making. | **Participants**: Patients and surgeons  **Sample size**: *N* = 18 patients; *N* = 8 surgeons  **Gender**: 50% male (patients), 63% male (surgeons)  **Age range**: Patients: *M* = 72.6, range: 64–89 years ; Surgeons: *M* = 43.8, range: 36–51 years  **Cancer localization**: rectal cancer  **Cancer grade**: N/A  **Treatment**: N/A  **Presence of a caregiver**: N/A | Qualitative inductive content analysis | In the surgical consultation, surgeons focused on communicating details of potential treatment pathways while patients sought information around prognosis, functional changes, and long term recovery. While surgeons often laid groundwork for shared decision-making, patient goals were not always clarified.  Patients responded by expressing confusion and asking for clarification.  When surgeons initiated discussion of an ostomy outside of their technical description, patients typically responded immediately and, at times, emotionally:“Oh no!.. Oh my god!”  Patients typically inquired about prognosis during the discussion of treatment options. Some asked explicitly about life expectancy with or without treatment, while others asked questions like,“what happens if we do nothing?”  Patients and families sought additional context, inquiring how the effects of surgery and recovery will impact their lives, including the ability to resume hobbies or return to work.  Patients shared both concrete and nebulous concerns about living with an ostomy. When patients described speci c concerns about managing or living with a stoma, surgeons responded with advice or recommendations. For some patients, the mere discussion of a stoma prompted a strong emotional reaction. When patients reacted negatively and nonspeci cally to a stoma, surgeons emphasized the stoma's necessity, without addressing the patient's emotional state.  Surgeons often presumed the patient's goal, if not directly expressed, was “getting rid of the cancer”. Without exploring patient statements and any contradictions therein, and with the uncertainty inherent in early consultations with incomplete clinical information, patient priorities imprecisely informed treatment plans. |
| [80] | Goss et al., 2015, Italy | N/A | Observational and quantitative study | Mixed methods, with audio-recorded consultations and questionnaires | To explore the frequency and type of questions asked by Italian breast cancer patients during their first oncological consultation and to explore associated patient and consultation characteristics. | **Participants**: Patients and oncologists  **Sample size**: *n* patients = 95; *n* oncologists = 3  **Gender**: 100% female (patients); 33% male (oncologists)  **Age range**: Patients: *M* = 58 years; range: 31–75 years; oncologists: N/A  **Cancer localization**: Breast  **Cancer grade**: Stage I, II, III **Treatment:** Lumpectomy  **Presence of a caregiver**: Yes (69%) | Sociodemographic and clinical data  Consultation characteristics  Physician–patient communication  Patients’ satisfaction | The average consultation duration was 34 min (± 12.5, range 12–72 min). Patients asked an average of 17.6 questions.  The topics were mostly related to illness management, particularly treatment, and administrative procedures. Contrastingly, patients asked fewer questions about prognosis, prevention, and etiology.  Overall, 59% of patients preferred a shared approach, 7% an active or very active role, and 34% a passive or very passive role.  Regarding the sociodemographic data, a greater number of questions were associated with being employed than not being employed; a greater number of questions about illness management, particularly the side-effects of radiotherapy, were related to a younger age.  Regarding the consultation, questions also increased with consultation duration, particularly those about illness management and administrative topics. Thirty-three consultations took less than 30 min, with an average of 13 questions asked by patients, while 37 consultations took more than 30 min, with an average of 21 questions. Patients who were prescribed chemotherapy asked more questions than those without to find out more about the procedure of chemotherapy. The longer patients had known their diagnosis, the more questions they asked about prevention. Question asking also increased when the oncologist correctly guessed the patient’s preferred role in the decision-making process. |
| [72] | Hack et al., 2010,  Canada | The Canadian Breast Cancer Research Alliance; the National Cancer Institute of Canada, with funds from the Canadian Cancer Society; the Sociobehavioral Cancer Research Network | Observational and quantitative study | Mixed methods, with audio-recorded consultations and questionnaires | To explain the content of primary adjuvant breast oncology consultations and to examine the predictive relationships between patient and oncologist consultation factors and patient satisfaction with communication | **Participants**: Patients and oncologists  **Sample size**: *N* patients = 172; *N* oncologists = 37  **Gender**: 100% female (patients)  **Age range**: Patients: *M* = 56 (*SD* = 12.9)  **Cancer localization**: Breast  **Cancer grade**: < 0, 1–3  **Treatment**: Lumpectomy, modified radical mastectomy, both treatments, missing, chemotherapy, hormone therapy  **Presence of a caregiver**: Yes (61.6%) | The content and mode of exchanges between patients and physicians (using the Medical Interaction Process System)  Patient behavior relative to anxiety, hostility, and nervousness | The total number of utterances for the 172 consultations was 73,403 (patient—19,625 utterances [26.7%]; oncologist—53,778 utterances [73.3%]).  Biomedical content categories were predominant in the consultations, accounting for 88.3% of all utterances, followed by administrative (5.9%) and psychosocial (5.8%) utterances.  It was most common for oncologists to lead the consultations and for patients to follow in the same content category; therefore, the percentages of content utterances per category were, unsurprisingly, similar for oncologists and patients.  The most common mode for patients was “registers information” (46.8 utterances per consultation; 41% of patient utterances), followed by “gives information” (39.7 utterances per consultation; 34.8% of patient utterances).  The average number of question utterances per consultation was 6.7 for patients and 10.3 for oncologists (a question typically consists of one to two utterances, so the true number of questions asked by patients and oncologists was lower than the mean utterance count). |
| [90] | Hamel et al., 2021, USA | National Cancer Institute | Observational study | Quantitative, with video-recorded consultations | To examine the dynamic interaction and behavioral convergence and divergence between Black cancer patients and their oncologists when discussing cancer treatment | **Participants**: Patients and physicians **Sample size**: *N* patients = 74; *N* physicians = 15 **Gender**: 92% female (patients); 47% female (physicians) **Age range**: Patients: *M* = 58.2 (*SD* = 10.5); physicians: *M* = 45.8 (*SD* = 11.6) **Cancer localization**: Breast, colorectal, lung **Cancer grade**: N/A **Treatment**: N/A **Presence of a caregiver**: N/A | Frequency of facial behaviors (smiles, eye gaze), body behaviors (lean, orientation, openness), and paraverbal behaviors (talk time, word count, interruptions, laughter, continuers) | Nonverbal behaviors that were most influenced by other nonverbal behaviors from one time point to the next were physician and patient smiling, physician gaze, patient continuers, and physician and patient interruptions.  Nonverbal behaviors that had the most influence on other nonverbal behaviors from one time point to the next were physician and patient gaze, physician and patient leaning in, physician interruption, and patient laughter. The results for patient talk time, physician gaze, and physician interruption revealed that as the frequency of a particular behavior increased, other behaviors accumulated to decrease it; if the frequency of a behavior decreased, the others accumulated to increase it, suggesting a regulatory subsystem. Several positive relationships between behaviors were found, including patient and physician smiling, gazing, and laughter; patient leaning in and physician smiling; patient interruptions and physician orientation to the patient; patient and physician interruptions. Negative relationships were found between patient leaning in and physician talk time, patient openness and physician gaze, and patient gaze and physician openness. |
| [68] | Henry et al.,  2015, USA | The Blue Cross Blue Shield of Michigan Foundation | Randomized controlled trial, observational and quantitative study | Quantitative, with audio-recorded consultations | To examine the overall structure of visits and how patients and physicians transition between communication activities during visits in which patients are newly diagnosed with prostate cancer | **Participants**: Patients and physicians  **Sample size***:* *N* patients = 40; *N* physicians = 18  **Gender**: 100% male (patients); 73.3% male (physicians)  **Age range**: Patients: *M* = 63.6 (*SD* = 5.0); physicians: *M* = 28.8 (*SD* = 2.0)  **Cancer localization**: Prostate  **Cancer grade**: Early-stage  **Treatment**: N/A  **Presence of a caregiver**: Yes (20%) | Empiric discourse analysis: functions and organization of language (e.g., duration, talk turn) | Activities and transitions during the opening sequence were usually brief, were similar across visits, and involved minimal patient speech. Patient speech during these transitions was mostly confined to backchannels.  Patient speech increased notably after the opening sequence: Transitions after the opening sequence included much more patient speech; this increase was due to an increase in both physicians’ eliciting patient talk and in patient questions.  During two visits, patients attempted to initiate transitions out of options talk. During both visits, patients attempted to transition away from options talk, but physicians did not cooperate. During two of these transitions, patients showed confusion about treatment options, and physicians transitioned back to options talk to address this confusion. During two other instances, patients transitioned to decision talk before the physician could start options talk. During both these instances, the physician quickly directed the discussion back toward options talk.  A few patients seemed unclear about the overall visit purpose and their role in the decision-making process. |
| [55] | Heyn et al., 2013, Norway | Research Council of Norway | Observational and quantitative study | Randomized controlled trial, with audio-recorded consultations | To test the effect of the choice of an interactive tailored patient assessment on the number and types of symptoms discussed during consultations with cancer patients, the active participation of patients during consultations with physicians, and the responses of physicians | **Participants**: Patients and physicians  **Sample size**: *N* patients = 193  **Gender**: 32% female (patients)  **Age range**: Patients: *M* = 49.07 (*SD* = 15.62), range: 18–80 years  **Cancer localization**: Lymphoma, multiple myeloma, leukemia, testicular  **Cancer grade**: N/A  **Treatment**: N/A  **Presence of a caregiver:** N/A | Type of utterance (i.e., medical, therapeutic, lifestyle, psychosocial, other)  The person who initiated each utterance (i.e., patient or clinician) | The mean of patient utterances was 51.3 (*SD* = 26.6, median = 47) during the total of 193 consultations. The patients in the intervention group had significantly more utterances in total compared with the patients in the control group.  When dividing the utterances into the five categories, only significantly more utterances in the therapeutic category were found.  No group differences were detected in the other categories (medical, lifestyle, psychosocial, and other).  Concerning utterances initiated by the patient, no group differences were retrieved in patients’ active participation. Patients asked significantly more closed-ended questions in the intervention group.  The patients who reported higher negative affects prior to the consultation presented a higher total number of utterances (*p* < .01) and, when dividing the utterances into the five categories, as defined by the Roter interaction analysis system, a higher number of utterances in the psychosocial category (*p* < .001).  The patients with more negative affects prior to the consultation initiated significantly more utterances than those with few negative affects (*p* = .001), and older patients initiated significantly more utterances than younger patients (*p* < .001). |
| [61] | Ishikawa et al., 2002a, Japan | Japanese Ministry of Education, Science, Sports, and Culture | Observational and quantitative study | Mixed methods, with audio-recorded consultations and questionnaires | To describe the characteristics of patient–physician communication during a Japanese cancer consultation, as well as to examine the relation of this interaction with patient satisfaction | **Participants**: Patients and physicians **Sample size***:* *N* patients = 140; *N* physicians = 12 **Gender**: 40% male (patients) **Age range**: Patients: *M* = 58.8 (*SD* = 11) **Cancer localization**: N/A **Cancer grade**: N/A **Treatment**: N/A **Presence of a caregiver**: Yes (*N* = 40) | Sociodemographic data Duration of the consultation The presence of family The number of utterances from members of the communication | A major part of the interaction concerned information giving among both the physicians and the patients; this consisted of 35% and 34% of their communication, respectively. Physicians asked nearly twice as many questions as patients, including open-ended and closed-ended questions. Patients made twice as many positive utterances as physicians.  The mean patient verbal dominance ratio was 0.99 (*SD* = 0.25), indicating that physicians and patients talked almost equally during consultations. On the other hand, the mean ratio of psychosocial to biomedical exchanges was 0.22 (*SD* = 0.22), and the patient centeredness ratio was 0.40 (*SD* = 0.24). The psychosocial exchange ratio was thus positively associated with the patient verbal dominance ratio. |
| [76] | Ishikawa et al., 2002b,  Japan | Japanese Ministry of Education, Science, Sports, and Culture | Observational and quantitative study | Mixed methods, with audio-recorded consultations and questionnaires | To explore the interaction between patient–physician communication in Japanese cancer consultations while considering the influence of patient and consultation characteristics | **Participants**: Patients and physicians  **Sample size**: *N* patients = 129; *N* physicians = 12  **Gender**: 59.7% female (patients)  **Age range**: Patients: *M* = 58.3 (*SD* = 11.1)  **Cancer localization**: N/A  **Cancer grade**: N/A  **Treatment**: N/A  **Presence of a caregiver**: Yes (*N* = 36) | Sociodemographic data Patient behaviors: information giving, question asking as information-seeking behavior, and emotional expression as psychosocial disclosure  Duration of the consultation  The presence of family  The number of utterances from members of the communication | A major part of the interaction was information giving among both the physicians and the patients, which comprised 35 and 33% of their communication, respectively.  Physicians asked nearly twice as many questions as patients, including both open-ended and closed-ended questions.  Patients made twice as many positive utterances as physicians (about 40% of their total utterances in the consultation on average).  The proportion of patient information giving was greater for the patients with a poorer physical status. The proportion of patient question asking was greater when patients were younger and the consultation was longer.  The global affect score was positively related to patient question asking, although physician facilitation was not significantly associated therewith.  The proportion of patient emotional expression was greater when patients were female and accompanied by their family. It was not significantly associated with physician emotional responsiveness, while it tended to have a positive association with the global affect score. |
| [10] | Leppin et al., 2018, USA | National Center for Complementary and Integrative Health (from the National Institutes of Health) | Observational study | Mixed methods, with audio-recorded consultations | To identify possible factors that could predict patient–physician concordance in the perception of whether any cancer care decisions are made during oncology encounters | **Participants**: Patients and providers **Sample size***:* *N* patients = 128 **Gender**: 55% female **Age range**: *M* = 62, range: 22–84 years **Cancer localization**: Brain, breast, gastrointestinal, genitourinary, gynecological, head or neck, lung, melanoma, sarcoma, unknown **Cancer grade**: Initial diagnosis, early initial treatment, mid-initial treatment, post-treatment or survivorship, recurrence or on treatment, end-stage **Treatment**: N/A **Presence of a caregiver**: N/A | Communication behaviors that might account for concordance and the degree of patient involvement in decision-making | Patients were major contributors to decisions in 23% of cases.  Patients were noted to have no participation in 9% of all cancer care decisions made.  The level of patient involvement was significantly associated with the type of decision being made (chi-squared *p* < .0001): Patients were major contributors in 59% of all logistical decisions but did not participate in or agreed only with 60% of all medical management decisions. |
| [119] | Marino et al., 2023, Italy | Sapienza University of Rome | Observational study | Quantitative, with video-recorded consultations | To explore potential differences in patient-centered care practices in two types of medical encounters: oncology visits between Italian physicians and Italian patients and oncology visits between Italian physicians and foreign patients, as well as to verify whether the differences in patient-centered care depend on the type of visit and the presence or absence of companions | **Participants**: Patients, companions, and oncologists  **Sample size***:* *N* patients = 42; *N* oncologists = 8; *N* companions = N/A  **Gender**: 76,2% female (patients)  **Age range**: Italian women: *M* = 57; foreign women: *M* = 52; Italian men: *M* = 71; foreign men: *M* = 61  **Cancer localization**: Breast, gastrointestinal stromal tumors, gynecological, lung, head and neck, urological, neuroendocrine tumors, liver, lymphoma tumors  **Cancer grade**: N/A  **Treatment**: N/A  **Presence of a caregiver**: Yes (*N* = 20) | The extent to which and how each participant (i.e., doctor, patient, and companion, if present) participated in various communicative actions | Older oncologists, patients from foreign countries, and the presence of a companion during the visit were associated with more interruptions during medical encounters. The other variables, including the type of encounter, patient age, and patient sex, did not significantly contribute to the prediction of interruptions.  The presence of a companion and being a foreign patient were found to play a role only in relation to interruptions during the visit.  The interruptions experienced by older oncologists included requests for advice, comments, and comparisons from peers in the same specialty about other patients, consultations from other specialists (such as radiotherapists) for the ongoing treatment of shared patients, the secretary entering the room to ask for information and/or resolve issues with patients and/or appointments, the secretary entering the room to collect or return medical records, and a patient leaving the room. |
| [13] | Mitchell et al., 2018, Northern Ireland | Department for Employment and Learning | Qualitative study | Qualitative, with audio-recorded consultations | To examine the evolution of communication dynamics for patients taking oral chemotherapy by considering to what extent concordance is important during consultations | **Participants**: Patients, family members, and oncologists **Sample size**: *N* patients = 8; *N* family members = 11; *N* oncologists = 15  **Gender**: 62.5% female (patients) **Age range**: Patients: *M* = 58.6, range: 28–78 years **Cancer localization**: Colorectal **Cancer grade**: N/A **Treatment**: N/A **Presence of a caregiver**: Yes (*N* = 7) | Thematic analysis | Four themes related to the patient treatment journey were identified: autocracy, physiological concordance, holistic concordance, and silence. While patients appeared passive and spoke infrequently during their first appointment, they were highly satisfied with this style of communication. Patients perceived that healthcare professionals provided them with the knowledge to competently self‐administer capecitabine. The theme of “physiological concordance” was present in participating patient consultations from appointment two until the fourth or fifth appointment, a period of approximately three to four months. During these consultations, it was more common for patients to lead communication about their experiences with chemotherapy by, for example, starting communication with healthcare professionals, asking questions, and bringing conversations back to their own life. Patients and their family members expressed satisfaction with the communication they received from the healthcare team up until this point.  The theme of “holistic concordance” was present in participating patient consultations from around appointment five onwards, which was about months four to six of the patient journey. In addition to comprising biomedical aspects, this theme demonstrated how patients and families communicated about psychosocial issues of chemotherapy management. This dialogue occurred as expressing sadness, uncertainty, and anger about living with cancer; sexual dysfunction; hair loss; the adjustments required for returning to work in future. |
| [73] | O'Neill et al., 2021, USA | National Cancer Institute | Randomized controlled trial, observational and quantitative study | Mixed methods, with audio-recorded consultations | To examine the test- and treatment-related information discussed, as well as the quality of communication, during these clinical encounters | **Participants**: Patients and oncologists  **Sample size**: *N* patients = 46; *N* oncologists = 13  **Gender**: 100% female (patients); 30.7% male (oncologists)  **Age range**: Patients: *M* = 59.7 (*SD* = 11.2)  **Cancer localization**: Breast  **Cancer grade**: Stages I and II  **Treatment**: N/A  **Presence of a caregiver**: N/A | Sociodemographic characteristics  Patient clinical characteristics  Patient active participation | Elements of patient active participation were higher among patients with high recurrence scores than among those with intermediate or low recurrence scores.  Differences were not statistically significant across recurrence scores in models adjusting for the receipt of chemotherapy. This included the global rating of patient active participation (overall *M* = 3.5/5; *p* = 0.29). |
| [120] | Ong et al., 2000, Netherlands | Dutch Cancer Foundation | Observational and quantitative study | Randomized controlled trial, with audio-recorded consultations and questionnaires | To investigate the relationship between both oncologists’ and cancer patients’ communication and patient quality of life and satisfaction | **Participants**: Patients and oncologists **Sample size**: *N* patients = 96; *N* oncologists = 11  **Gender**: 83% female (patients) **Age range**: Patients: *M* = 53 (*SD* = 16.5) **Cancer localization**: Gynecological (vulva, cervix, corpus, ovary) or medically oncological (malignancy of the breast, bladder, skin, testis, liver, pancreas, esophagus, colon) **Cancer grade**: N/A **Treatment**: N/A **Presence of a caregiver**: N/A | Socio-emotional behaviors  Doctor and patient affects  Patient satisfaction with the consultation Global patient satisfaction with communication | Patients’ instrumental behaviors and their quality of life were unrelated.  Their social behaviors were moderately related to both their physical distress and their global quality of life at T2. Patients’ anger was moderately related to psychological distress, both at T1 and T2.  Patients’ anxiety was moderately related to their psychological distress, as well as to their global quality of life. A negative relation was found between patient question asking and their visit-specific satisfaction at T2.  Patients’ negative talk was negatively related to visit-specific satisfaction at both T1 and T2 and their global satisfaction at both follow-up points.  Patients’ directive statements, information giving, social behaviors, verbal attentiveness, and concern showing were unrelated to their satisfaction.  Patients’ anger was negatively related to visit-specific satisfaction at T2, whereas both patients’ interest and friendliness were positively related to their visit-specific satisfaction at T1 and T2.  Anger was also related to patients’ global satisfaction at T1.  Finally, patients’ anxiety, dominance, and signs of distress were unrelated to satisfaction. |
| [74] | Reese et al., 2019, USA | American Cancer Society; National Cancer Institute | Observational and quantitative study | Mixed methods, with audio-recorded consultations and questionnaires | To examine the prevalence and content of sexual health communication between breast cancer patients and physicians | **Participants**: Patients and clinicians  **Sample size**: *N* patients = 67; *N* clinicians = 7  **Gender**: 100% female (patients)  **Age range**: Patients: *M* = 56.6 (*SD* = 12.2)  **Cancer localization**: Breast  **Cancer grade**: Stages I–II, stages III–IIIC, stage IV  **Treatment**: On active treatment, completed adjuvant treatment, on hormonal therapy, off all treatment, surgery, lumpectomy, mastectomy, chemotherapy, radiation therapy, hormonal therapy  **Presence of a caregiver**: N/A | Sociodemographic characteristics  Medical data  Communication about sexual topics | Of the 22 visits with women reporting sexual problems, 10 visits (45%) contained sexual health communication, whereas the remaining visits for women reporting sexual problems (55%) did not have sexual health communication.  Women reporting sexual problems were no more likely to have sexual health communication in their visits as those not reporting sexual problems.  During nine visits, a sexual health discussion occurred, either because the patient responded with anything other than outright denial of a concern or because the patient (or a third party) raised the topic and directed the discussion. In both cases, the patients indicated no need for further discussion by denying vaginal dryness outright: No further discussion ensued.  There were eight clinic visits with women reporting sexual problems who had in-depth discussions. During these discussions, patients gave an affirmative indication of a problem in response to a clinician’s query or raised the issue themselves, and a discussion ensued. The clinician or patient each initiated the discussion during four (50%) encounters. Specifically, the patient initiated the discussion twice by mentioning negative feelings about breast appearance post-surgery. During one visit, a patient initiated the discussion by referring to an established problem of vaginal dryness and the potential use of hormonal treatment for this discomfort. Finally, during one visit, the patient initiated the discussion on behalf of her husband, discussing whether sexual activity was safe while undergoing chemotherapy.  There were nine clinic visits with women not reporting sexual problems who nevertheless engaged in an in-depth discussion of sexual health with their clinicians. The clinician initiated the sexual health communication during five of these visits (56%), whereas the patient initiated the discussion during three visits, and the patient’s husband initiated the discussion during one visit.  During two visits, the patient initiated the sexual health discussion by bringing up negative feelings about the appearance of her breasts. During one visit, in response to mention of a study on intimacy, the patient stated that “there is no intimacy at my house,” thereby raising the topic of sexuality by indicating a lack of sexual activity in her relationship. Finally, during one visit, the husband initiated the discussion by asking whether sexual activity was safe while his wife was undergoing chemotherapy. |
| [63] | Robinson et al., 2016,  USA | National Cancer Institute | Qualitative study | Qualitative, with video-recorded consultations | To identify the content of questions that US breast cancer patients ask surgeons during treatment decision-making consultations | **Participants**: Patients and surgeons  **Sample size***:* *N* patients = 132  **Gender**: 100% female  **Age range**: *M* = 61.2 (*SD* = 12.6)  **Cancer localization**: Breast  **Cancer grade**: Stages 0–1  **Treatment**: N/A  **Presence of a caregiver**: Yes (82.6%) | Thematic analysis | Theme 1: Asks for a Description of the Surgical Procedures of Lumpectomy or Mastectomy: Questions from this theme were asked in 50.76% of all consultations (67/132), and when this theme was raised, patients asked an average of 2.76 thematic questions (i.e., theme density).  Theme 2: Asks About the Type of Surgical Procedure Needed or More or Less Appropriate or Recommended: Questions from this theme were asked in 48.48% of all consultations (64/132), and when this theme was raised, patients asked an average of 1.83 thematic questions.  Theme 3: Asks About the Date or Timeframe When Surgery Could Occur: Questions from this theme were asked in 44.70% of all consultations (59/132), and when this theme was raised, patients asked an average of 1.42 thematic questions. This theme represented questions about when surgery could be conducted.  Theme 4: Asks for a Description of Procedures Involving Testing or Removing of Lymph Nodes: Questions from this theme were asked in 39.39% of all consultations (52/132), and when this theme was raised, patients asked an average of 2.29 thematic questions. This theme represented questions about the procedures of testing and removing lymph nodes.  Theme 5: Asks About the Surgical Recovery Process or Timeframe: Questions from this theme were asked in 39.39% of all consultations (52/132), and when this theme was raised, patients asked an average of 2.08 thematic questions.  Theme 6: Asks About the Spread of Cancer to Lymph Nodes or the Body: Questions from this theme were asked in 31.06% of all consultations (41/132), and when this theme was raised, patients asked an average of 1.98 thematic questions.  Theme 7: Asks for a Description of the Procedure Involving Radiation Therapy: Questions from this theme were asked in 27.27% of all consultations (36/132), and when this theme was raised, patients asked an average of 2.06 thematic questions.  Theme 8: Asks About the Severity of the Cancer or Tumor and/or Its Characteristics: Questions from this theme were asked in 25.76% of all consultations (34/132), and when this theme was raised, patients asked an average of 1.26 thematic questions. This theme involved evaluative questions about the “severity” of the cancer or tumor itself.  Theme 9: Asks About the Need or Rationale for Chemotherapy: Questions from this theme were asked in 24.24% of all consultations (32/132), and when this theme was raised, patients asked an average of 1.78 thematic questions.  Theme 10: Asks About the Type of Tumor Involved: Questions from this theme were asked in 24.24% of all consultations (32/132), and when this theme was raised, patients asked an average of 1.59 thematic questions. This theme involved questions about the specific type of tumor involved.  Theme 11: Asks About the Need or Rationale for Radiation: Questions from this theme were asked in 24.24% of all consultations (32/132), and when this theme was raised, patients asked an average of 1.38 thematic questions.  Theme 12: Asks for a Description of the Procedure Involving Reconstruction: Questions from this theme were asked in 23.48% of all consultations (31/132), and when this theme was raised, patients asked an average of 2.52 thematic questions.  Theme 13: Asks About Hormone Therapy and Its Rationale: Questions from this theme were asked in 21.21% of all consultations (28/132), and when this theme was raised, patients asked an average of 1.88 thematic questions.  Theme 14: Asks About Their Hormone-Receptor and HER2 Status: Questions from this theme were asked in 21.21% of all consultations (28/132), and when this theme was raised, patients asked an average of 1.61 thematic questions.  Theme 15: Asks About Recurrence and Its Likelihood or Detection: Questions from this theme were asked in 21.21% of all consultations (28/132), and when this theme was raised, patients asked an average of 2.04 thematic questions. |
| [64] | Rodriguez et al., 2010, USA | VA HSR&D Merit Review Entry Program Award; National Cancer Institute | Qualitative study | Qualitative, with audio-recorded consultations | To describe the content and frequency of communication about health-related quality of life during outpatient encounters between oncologists and their advanced cancer patients | **Participants**: Patients and oncologists  **Sample size***:* *N* patients = 70; *N* oncologists = 37  **Gender**: 47% male (patients); 78% male (oncologists)  **Age range**: Patients: *M* = 59.3 (*SD* = 12.4); oncologists: *M* = 44.3 (*SD* = 7.6)  **Cancer localization**: Hematologic, breast, lung, colon or gastrointestinal, brain, other  **Cancer grade**: N/A  **Treatment**: Chemotherapy, endocrine therapy, radiotherapy, surgery, no treatment, other  **Presence of a caregiver**: N/A | Content analysis to code and describe the content and frequency of communication about health-related quality of life during encounters. | All 73 encounters included a discussion of health-related quality of life.  In 17 encounters (23%), the first mention of health-related quality of life was by the patient, typically in relation to symptoms (24%) or physical (18%), functional (18%), psychological (18%), or other concerns (24%). Less common were discussions about the impact of the disease or treatment on a patient’s ability to work (17%).  Emotions, mental health, and psychological health-related quality of life were introduced into the conversations more frequently by patients than providers (59 vs. 41%) and occurred in only 9% of the encounters.  When psychological concerns were discussed, they centered on topics of cognitive functioning and decision-making ability (18%), the onset of depression (16%), increases in fear (12%), increases in stress (10%), and negative body image related to hair loss, changes in weight, or less attractive general appearance (10%).  Spiritual health-related quality of life was introduced into conversations more frequently by patients than providers (75 vs. 25%) and discussed in only four encounters (1% of all encounters).  A few patients mentioned an increase in praying or Bible reading to help them cope, and one mentioned the adoption of tai chi to help him cope. |
| [59] | Roter et al., 2016, USA | National Cancer Institute of Complementary and Alternative Medicine | Observational and quantitative study | Mixed methods, with audio-recorded consultations | To describe discussions about the use of complementary alternative medicine during oncology visits, the communication patterns that facilitate these discussions, and their association with visit satisfaction | **Participants**: Patients and providers  **Sample size***:* *N* patients = 327  **Gender**: 59% female  **Age range**: *M* = 61.4 (*SD* = 13.5), range: 22–90 years  **Cancer localization**: Brain, breast, gastrointestinal, genitourinary, gynecological, head or neck, lung, melanoma, sarcoma, other  **Cancer grade**: Initial diagnosis, early treatment, mid-treatment, remission, recurrence, end-stage  **Treatment**: N/A  **Presence of a caregiver**: Yes (67%) | Duration of the session in minutes  Contributions of each speaker to the total dialogue (i.e., the sum of all patient statements, the sum of all patient and companion statements, and the sum of all clinician statements)  Measures of verbal dominance constructed as the ratio of total patient statements to clinician statements (excluding the patient’s companion) and the ratio of the sum of patient and companion statements relative to clinician statements  The nature of the exchange, the point in the visit at which the discussion took place, and its duration in seconds Sociodemographic and self-reported health status variables | More than half of the first complementary and alternative medicine conversations of the visit *(n* = 22; 61%) were initiated by the patient, while 12 (33%) were clinician-initiated. Only two of the initial complementary and alternative medicine conversations were companion-initiated.  During the 29 visits in which there were multiple complementary and alternative medicine-related exchanges, clinicians initiated slightly more than half (*n* = 15; 52%), patients initiated 10 (34%), and companions initiated four of the exchanges.  The duration of the discussion was not related to the initiator; patient-initiated discussions averaged 49 s, and those initiated by clinicians averaged 47 s.  Among the patients who discussed complementary and alternative medicine during the visit, 46% (*n* = 17) were in early or mid-stages of treatment, 38% (*n* = 14) were in remission, and 16% (*n* = 6) were experiencing recurrence or end-stage disease (Chi-squared = 6.0; *p* < 0.05).  Discussions were initiated during the history segment (76%) or counseling segment (19%) of the visit. Only three discussions were observed during the opening or physical exam.  Patients were significantly more verbally active during visits with complementary and alternative medicine compared with non-complementary and alternative medicine visits; clinician and companion contributions to the visit dialogue did not differ in terms of the presence of a complementary and alternative medicine discussion.  Visit dialogue was less verbally dominated by clinicians in complementary and alternative medicine compared to non-complementary and alternative medicine visits.  Patients disclosed significantly more psychosocial and lifestyle information during visits that included a complementary and alternative medicine discussion, and clinicians conveyed significantly more psychosocial information to their patients during these visits.  Medical information exchanges did not differ relative to complementary and alternative medicine discussions. |
| [77] | Schellenberger et al., 2022, Germany | German Cancer Aid | Observational and quantitative study | Mixed methods, with video-recorded consultations and questionnaires | To investigate the number of questions and negative emotions (cues or concerns) that breast and/or gynecological cancer patients and companions express during their case discussion in multidisciplinary tumor conferences, to which topics the emotions are related, and which patient- and context-related characteristics determine patients’ communicative behavior | **Participants**: Patients and providers  **Sample size***:* *N* patients = 82  **Gender**: 100% female  **Age range**: *M* = 59 (*SD* = 11.2)  **Cancer localization**: Breast and/or gynecological  **Cancer grade**: Stage 0, I, II, III, IV, missing  **Treatment**: N/A  **Presence of a caregiver**: Yes, but not specified | Sociodemographic data  Expressed questions and cues or concerns of patients and companions  Emotional expressions of patients and companions | Patients asked 460 questions (76%), and companions asked 147 questions (24%), for a total of 607 questions. The questions were divided into 391 informational questions, 180 confirmatory questions, 18 repair questions, and 18 rhetorical questions.  A total of 230 cues or concerns (average 2.8 per case, median: 2, range: 0–24) were identified in the case discussions, 198 (86%) expressed by patients and 32 (14%) by companions. Most of the cues or concerns were patient-elicited (209, 91%). No cues or concerns were expressed in 11 case discussions (13%). Among the cues, neutral, salient expressions occurred most frequently (73%). Cues were most frequently expressed when discussing the treatment in general (64 cues or concerns, 28%), followed by cues or concerns linked to the diagnosis (46 cues or concerns, 20%) and cues or concerns linked to possible side-effects of the treatment (43, 19%).  Fear of progression or recurrence was linked to 12% of cues or concerns (28 cues or concerns) and comorbidities to 11% (25 cues or concerns).  Only 10 of the 230 cues or concerns (4%) were linked to general uncertainty or doubt and self-reproach, and nine (4%) were linked to the burden or risk for relatives.  Concerning the ability to work, five cues or concerns were expressed (2%).  Significantly more questions about the need for psychological support (4.259, with *p* = 0.001) and being accompanied (3.398, with *p* = 0.006) were asked during case discussions by patients and companions.  Patients expressed fewer questions in hospital 2 (compared with hospital 1: −5.027, *p* = 0.001) and in hospital 4 (compared with hospital 1: −5.728, *p* = 0.023).  In model 2, no significant predictors were found for the number of cues or concerns expressed. |
| [65] | Sherlock et al., 2019, England | Health Foundation | Qualitative study | Qualitative, with audio-recorded consultations | To examine the nature of discourse when patients ask physicians for a treatment recommendation during consultations when treatment decisions are shared, as well as to examine the strategies used by physicians in response | **Participants**: Patients and clinicians **Sample size***:* *N* patients = 25 **Gender**: 100% female **Age range**: N/A **Cancer localization**: Breast **Cancer grade**: Early-stage **Treatment**: N/A **Presence of a caregiver**: Yes, but not specified | Theme‐orientated discourse analysis | In consultations with eight of the 25 patients, there were moments when either the patient or their partner attempted to defer treatment decisions to the clinician, with six where the patient asked the clinician for a recommendation, and another two where the patient's partner asked.  One patient indicated her desire for the clinician to take decisional responsibility on five separate occasions during her two consultations. The patient was then given the opportunity to ask questions; this was generally the point during the consultation when the patient sought a treatment recommendation.  Patients anticipated the clinician's reluctance to give a treatment recommendation, even before they asked for it. |
| [60] | Siminoff et al., 2000, USA | N/A | Observational and quantitative study | Mixed methods, with audio-recorded consultations and questionnaires | To identify variables within the patient–oncologist communication pattern that impact patients' overall understanding of and satisfaction with breast cancer treatment | **Participants**: Patients and oncologists  **Sample size***:* *N* patients = 50  **Gender**: 100% female  **Age range**: *M* = 63.9 (*SD* = 7.3)  **Cancer localization**: Breast  **Cancer grade**: N/A  **Treatment**: N/A  **Presence of a caregiver**: N/A | Percentage of the total utterances spoken by the patient  Percentage of the total utterances spoken by the physician, coded as affective (i.e., emotional)  Total number of questions asked by the patient during the consultation | Measures of patient comprehension were found to be positively associated with patients who spoke more, asked more questions, or had oncologists who used fewer affective utterances. In general, patients who spoke more during the consultation tended to provide more accurate responses to questions about their comprehension of adjuvant therapy.  Patients who asked more questions were significantly more likely to correctly recognize that adjuvant therapy will not help all breast cancer patients (*p* < 0.05) and that physicians cannot always know if adjuvant therapy will help them (*p* < 0.05), compared to patients who asked fewer questions. These patients also tended to be more likely than patients who asked fewer questions to know that some patients live with surgery alone, recognize that patients cannot know for certain whether adjuvant therapy is needed, define adjuvant therapy correctly, or know that treatment does not necessarily cure everyone.  Patients whose oncologists used more affective utterances tended to exhibit lower degrees of comprehension than patients whose physicians used less affect.  Patients who asked more questions were marginally less likely to believe that their physician adequately explained the need for more treatment than patients who asked fewer questions. They also tended to be less satisfied with the information they received, the amount of time they spent with their oncologist, and their overall visit. They reported less satisfaction with the notion that their physician understood their treatment goals or was concerned about their fears.  No significant relationship or trend was identified between physicians' use of affect and patient satisfaction.  White patients were found to be more proactive during the consultation, being significantly more likely to speak more often (*p* < 0.01) and were able to answer more comprehension questions correctly *(p* < 0.01). White patients were also marginally more likely to exhibit lower overall satisfaction than non-White patients (*p* < 0.1). |
| [78] | Siminoff et al., 2006, USA | National Cancer Institute | Observational study | Mixed methods, with audio-recorded consultations | To examine whether patient characteristics are associated with patterns of communication between oncologists and breast cancer patients | **Participants**: Patients and oncologists  **Sample size***:* *N* patients = 405*;* *N* oncologists = 58  **Gender**: 100% female (patients); 67.2% male (oncologists)  **Age range**: Patients: < 60 = 44.2%, > 60 = 55.8%; oncologists: < 40 = 51.7%, > 40 = 48.3%  **Cancer localization**: Breast  **Cancer grade**: N/A  **Treatment**: Adjuvant therapy  **Presence of a caregiver**: N/A | The structure of cancer communications to determine communication patterns  Patient variables, consisting of the patient communicating biomedical information (e.g., family medical history, how the breast tumor was discovered) to the oncologist; the patient communicating psychosocial information (e.g., lifestyle, social, and economic information) to the oncologist; a count of all questions asked by the patient; patient attempts to build a relationship with the physician (e.g., social and positive talk); the patient engaging in a discussion about their emotional status or expressing how they are feeling about their situation; the patient instigating or being proactive in raising a subject, offering information, or asking a question | Overall, 65% of utterances were made by physicians (median number of utterances = 350) compared to 35% by patients (median number of utterances = 202).  White patients provided more biomedical information to their physicians than did non-White patients (median = 88.0 vs. 73.0, *p* < 0.05).  Younger patients averaged 226 utterances compared to older patients with 157 utterances (*p* < 0.01).  More biomedical discussions were observed with White compared to non-White patients (median = 191 vs. 142*,* *p* < 0.01) and patients with higher educational attainment (median = 206.5 vs. 165, *p* < 0.01).  Finally, higher income patients (median utterances = 227.5) were given more biomedical information than medium (median = 183.5) and low income (median = 152.5) patients (*p* < 0.01).  The number of patient questions varied by all sociodemographic variables. Younger patients asked more questions, as did those who were White, had more than a high school education, and earned a high or medium income compared to those who earned a low income (median = 25 and 22.0 questions vs. 14.0 questions, *p* < 0.01).  Patient proactive behavior, such as volunteering information to the physician unasked, was similarly related with all demographic predictors. The logistic regression found that race (OR = 2.48, 95% CI 1.43–4.30*;* *p* < 0.01), age (OR = 0.27, 95% CI 0.17–0.45; *p* < 0.001), and the interaction term between age and education (OR = 3.36, 95% CI 1.90–5.95; *p* < 0.001) remained significantly correlated to patients’ question-asking behaviors.  Both patients and physicians spent time trying to establish an interpersonal relationship with each other. Patients voiced more relationship-building utterances than their physicians, though. Patient median utterances relevant to relationship building were 67 compared to 58.5 for physicians. Patients differed in the number of relationship-building utterances by age (younger patient median = 75 vs. older patient median = 63, *p* < 0.01), education (higher level of education median = 76 vs. lower level of education median = 62.5, *p* < 0.01), and income (higher income median = 75.5 vs. middle income median = 73 and lower income median = 59*,* *p* < 0.01). The logistic regression examining patient relationship-building behavior revealed that educational level (OR = 1.81, 95% CI 1.21–2.71; *p* < 0.01) was a significant independent predictor. |
| [46] | Song et al., 2015, USA | Clinical and Translational Sciences Award; American Cancer Society; Urology Care Foundation | Randomized controlled trial, qualitative and observational study | Randomized controlled trial, with audio-recorded consultations | To describe patient–physician communication behaviors and patient involvement in treatment decision-making communication during consultation visits for localized prostate cancer | **Participants**: Patients and physicians  **Sample size***:* *N* patients = 52  **Gender**: 100% male  **Age range**: *M* = 60.2, *SD* = 7.1, range: 45–73 years  **Cancer localization**: Prostate  **Cancer grade**: LPCa stages T1a, T1b, T1c, T2a, or T2b  **Treatment**: N/A  **Presence of a caregiver**: N/A | Sociodemographic data  Communication behaviors, including information giving, clarifying or verifying, and seeking  Patient participation in communication during consultations | More than half of the patients engaged in information giving, clarifying or verifying, and seeking behaviors when discussing their health histories, survival or mortality, treatment options of surgery and radiotherapy, potential treatment impacts (e.g., urinary and sexual side-effects), and treatment preferences. Overall, 25% to 50% of the patients demonstrated these communication behaviors when discussing treatment options related to watchful waiting or active surveillance or hormonal therapy, treatment impact (complications, quality of life, bowel and hormonal side-effects), and the management of side-effects.  Patients with low and intermediate prostate cancer risk demonstrated similar participation patterns.  Patient participation ranged from low to high when discussing their health histories.  Patient participation was moderate when discussing cancer diagnoses and comorbid conditions but low when discussing current prostate cancer-related symptoms.  Patient participation was low when discussing survival and mortality issues.  Regarding treatment options, patient participation was moderate when discussing surgery and radiotherapy and low when discussing hormonal therapy and active surveillance or watchful waiting.  Patient participation was none or low when discussing potential treatment impacts.  Patient participation was low when discussing complications, quality of life issues, urinary and sexual side-effects, and the management of urinary and sexual side-effects, whereas hormonal and bowel side-effects and the management of these side-effects were not discussed.  Patient participation was low when discussing their treatment preferences. |
| [87] | Step et al., 2009, USA | Aging-Cancer Research Development Program; National Institutes of Health; Case Western Reserve University | Observational and quantitative study | Quantitative, with audio-recorded consultations and questionnaires | To explore the direct effects of oncologists' relational communication on an important aspect of patient-centered care, the involvement of patient communication, and the indirect effects on decision regret for adjuvant therapy after early-stage breast cancer surgery | **Participants**: Patients and oncologists  **Sample size***:* *N* patients = 180*;* *N* oncologists = 40  **Gender**: 100% female (patients)  **Age range**: Patients: *M* = 64 (*SD* = 8.9)  **Cancer localization**: Breast  **Cancer grade**: Early-stage (I–III)  **Treatment**: Adjuvant therapy following surgery  **Presence of a caregiver**: N/A | Type of communication (instrumental or relational)  Patient communication involvement | A significant association was found between oncologist relational communication and patient communication involvement *(p* = 0.001). Greater positive oncologist relational communication predicted greater patient communication involvement.  A significant association was found between patient communication involvement and patient decision regret *(p* = 0.025).  Significant predictors of greater patient communication involvement included slightly higher patient education, more oncologist confirmation, less oncologist directness, and greater oncologist inclusion.  Significant path relationships between oncologist instrumental communication and patient communication involvement included more talk about treatment and prognosis.  Concerning the decision regret outcome, a small effect was found for the influence of patient age and education on decision regret, but a larger effect was found for patient communication involvement. Patients who were more communicatively involved in adjuvant therapy discussions reported significantly less decision regret three months later. |
| [91] | Street et al., 2008, USA | Career Development Award; the Office of Research and Development, Health Services Research, and Development Service, Department of Veterans Affairs, from the Agency for Healthcare Research and Quality | Observational and quantitative study | Randomized controlled trial, with audio-recorded consultations and questionnaires | To compare the communication of unaccompanied patients, accompanied patients, and companions during lung cancer consultations | **Participants**: Patients, physicians and companions **Sample size**: *N* = 132 (patients); *N* = 84 (companions) **Gender**: N/A **Age range**: Non-accompanied patients: *M* = 65.4; accompanied patients: *M* = 67.1 **Cancer localization**: Lung **Cancer grade**: N/A **Treatment**: N/A **Presence of a caregiver**: Yes (*N* = 84) | Sociodemographic data Verbal behavior coding from patients, companions, and physicians | The duration of the consultation (as measured by the total number of patient + companion + physician utterances) did not differ depending on whether a companion was present, nor were there differences in to what extent the physician controlled the conversational floor.  Unaccompanied patients expressed more negative affect than did accompanied patients and their companions. Accompanied patients on average talked more, were more active participants generally, and were more assertive than their companions. However, while companions held the floor proportionally less than accompanied patients, proportionally more of their talk was in the form of active participation than for accompanied patients. |
| [86] | Street et al., 2010, USA | American Cancer Society; National Institute of Mental Health; Houston Health Services Research and Development Center of Excellence at Michael E. DeBakey; VA Medical Center | Randomized controlled trial and observational study | Randomized controlled trial, with audio-recorded consultations | To examine the effect of a theoretically grounded, tailored education coaching intervention to help patients discuss their pain-related questions, concerns, and preferences with physicians more effectively | **Participants**: Patients and physicians  **Sample size***:* *N* patients = 148  **Gender**: 78% female for the tailored education coaching group; 84% female for the control group  **Age range**: *M* = 59.8 for the tailored education coaching group; *M* = 56.6 for the control group  **Cancer localization**: Lung, breast, prostate, head and neck, esophageal, colorectal, kidney, bladder, or melanoma  **Cancer grade**: Advanced or disseminated cancer  **Treatment**: N/A  **Presence of a caregiver**: N/A | Patients’ active participation  Pain-specific active participation | Patients in the two experimental groups did not differ in their total active participation (means = 14.77 and 14.75 utterances for the tailored education coaching and control groups, respectively). However, patients in the tailored education coaching intervention did display more pain-specific active participation (mean = 6.21 utterances) than did the control group (mean = 4.63) (*p* = .008).  In the multivariate analysis, patients in the tailored education coaching and enhanced usual care control groups did not differ in their total active participation. Rather, more active patients were younger, reported more pain at baseline, and interacted with doctors who encouraged participatory decision-making. However, patients in the tailored education coaching group did communicate more about pain-related issues compared to those in the control group, even when accounting for other factors that could have influenced patient participation.  The adjusted mean difference between the two groups was 1.51 utterances, indicating that the tailored education coaching group had 31% more questions, acts of assertiveness, and expressed concerns about pain than did the control group.  Patients who more actively discussed pain concerns had higher baseline pain and interacted with physicians using more participatory decision-making. |
| [67] | Street et al., 2014, USA | American Cancer Society; the Houston VA Center for Innovations in Quality, Effectiveness, and Safety | Randomized controlled trial, qualitative and quantitative study | Quantitative, with audio-recorded consultations | To test a pathway through which a tailored pain management education coaching intervention can contribute to better cancer pain control through the effects of patients’ pain communication on physicians’ prescription of pain medication | **Participants**: Patients and physicians **Sample size***:* *N* patients = 135 **Gender**: 82% female **Age range**: *M* = 58.2, range: 32–80 years **Cancer localization**: N/A **Cancer grade**: Advanced or disseminated cancer **Treatment**: N/A **Presence of a caregiver**: Yes (22%) | Patients’ active communication about pain | Older patients were less likely to report a change in pain medication (*r* = .17, *p* < .05). Patients’ pain-specific active communication was significantly correlated with changes in pain medication (*r* = .49, *p* < .0001) but only marginally with improvements in pain at six weeks (*r* = .15, *p* = .08).  Changes in pain medication were significantly predicted by having participated in the coaching–education intervention, higher baseline pain, and a younger age.  Patients were more likely to get a change in pain management if they communicated more actively about pain-related issues. |
| [56] | Takeuchi et al., 2011,  England | Cancer Research UK; Bramall Research Fellowship | Randomized controlled trial, qualitative and quantitative study | Randomized controlled trial, with audio-recorded consultations and questionnaires | To examine how patient-reported feedback impacts patient–physician communication over time to gain a better understanding of how it may influence patient care | **Participants**: Patients and physicians  **Sample size***:* *N* patients = 198*;* *N* physicians = 28  **Gender**: 78.7% female (patients); 60.7 male (physicians)  **Age range**: Patients: *M* = 56, range: 23–85 years; physicians: *M* = 33.5, range: 26–51 years  **Cancer localization**: Gynecological, breast, renal, bladder, sarcoma, melanoma, other  **Cancer grade**: Disease free, localized, or metastatic  **Treatment**: Chemotherapy or biologic therapy  **Presence of a caregiver**: Yes, but not specified | Content analysis to note the discussion of symptoms or psychosocial function | Patients in the intervention arm discussed more symptoms during consultations than those in the attention-control and control arms. The increase in symptom discussions was largest the first time patients reported outcomes to oncologists and was maintained over time. There were no differences between arms for the discussion of functions, and no time effect was observed. Of the identified covariates, only diagnosis remained significant in the functions model. Patients with melanoma discussed more, and patients with bladder cancer discussed fewer, functional issues than patients with other diagnoses. However, the numbers of patients with these cancers were small.  The discussion of symptoms and functions was predominantly initiated by patients or relatives, except for dyspnea and bowel habits.  A substantial proportion of patients (32% to 73%) denied the presence of symptoms listed; only a minority of patients reported severe symptoms (3% to 9%). Fatigue was an exception, with 21% of patients reporting severe fatigue. A large proportion of patients expressed poor role and social functioning (32% and 40%, respectively), whereas physical and cognitive functions were generally good (poor functioning reported in 12% and 4%, respectively). Patients reporting severe symptoms were more likely to discuss those symptoms. Severity was predictive of discussions about dyspnea *(p < .*01; covariate: baseline discussion) and pain (*p* < .001; covariate: extent of disease) during all three clinic encounters. The severity of fatigue (covariates: baseline discussion, patient’s sex, oncologist’s sex), nausea or vomiting (covariate: baseline discussion), anorexia (covariates: baseline discussion, age), and insomnia (covariates: baseline discussion, emotional functioning score, depression score) were significant predictors of discussion at two of the three clinic encounters. The constipation score was a significant predictor for the discussion of bowel habits, but only at the first consultation (*p* < .001; covariate: baseline discussion), but there was a positive trend in the second and third consultations (*p* < .015 and *p* < .030, respectively). There was no significant effect of the arm on whether a specific symptom was discussed. |
| [84] | Tang et al., 2018, USA | Behavioral Cooperative Oncology Group; Center for Symptom Management; Walther Cancer Foundation | Qualitative study | Qualitative, with audio-recorded consultations | To develop a typology to describe patterns of interactions between advanced pancreatic cancer patients, their caregivers, and healthcare providers concerning symptoms and symptom management | **Participants**: Patients, caregivers, oncologists, and nurses  **Sample size**: *N* patients = 37; *N* caregivers = 34*;* *N* oncologists = 37*;* *N* nurses = 9  **Gender**: N/A  **Age range**: N/A  **Cancer localization**: Pancreatic  **Cancer grade**: Advanced stages  **Treatment**: N/A  **Presence of a caregiver**: Yes (*N* = 34) | Thematic analysis | In 31 of the healthcare encounters, an interaction pattern was labeled a collaborative interaction. Collaborative interactions were those in which patients or caregivers and providers worked together to discover the nature of a symptom or a course of action.  In 28 of the healthcare encounters, an interaction pattern was labeled an explanatory interaction. Explanatory interactions were those in which providers gave factual information to patients and caregivers.  In 20 of the healthcare encounters, an interaction pattern was labeled an agentic interaction. Agentic interactions were those in which patients and/or caregivers were particularly vocal during discussions with providers and/or very involved in making treatment decisions.  In 16 of the healthcare encounters, an interaction pattern was labeled a checklist interaction. Checklist interactions were interactions in which providers asked a series of questions in rote fashion, as if they were “going down a list.”  In 14 of the healthcare encounters, an interaction pattern was labeled a cross-purpose interaction. Cross-purpose interactions were those in which patients and/or caregivers and providers seemed to have differing purposes and failed to acknowledge the remarks of the other. Often, the providers changed the focus of the discussion without attending to the concerns expressed by the patients or caregivers. For example, one patient mentioned how his fatigue was negatively affecting his quality of life and questioned the value of chemotherapy.  In 12 of the healthcare encounters, an interaction pattern was labeled an empathic interaction. Empathic interactions were those in which providers showed, or attempted to show, that they understood and cared about the emotions or experiences of the patients and/or caregivers.  In six of the healthcare encounters, an interaction pattern was labeled an admonishing interaction. Admonishing interactions were those in which providers cautioned patients and caregivers against behaviors thought not to be in the patients’ best interests or gently scolded them for taking certain actions or voicing certain opinions.  In four of the healthcare encounters, an interaction pattern was labeled a diverging interaction. Diverging interactions were those in which providers and patients and/or caregivers, although discussing the same topic, expressed disagreement. Some of the disagreements were related to the meaning or importance of the patients’ symptoms. Others were related to treatment decisions. Some of these interactions seemed tense. |
| [47] | Timmermans et al., 2006, Netherlands | Dutch Cancer Society | Observational study | Non-randomized trial, with video-recorded consultations | To study whether cancer patients’ participation in radiotherapy consultations can be improved by specific communicative behaviors of the radiation oncologists | **Participants**: Patients and staff members **Sample size***:* *N* patients = 160 **Gender**: 57% female in the pre-training group, 48% female in the post-training group **Age range**: Pre-training group: *M* = 58.9 (*SD* = 13.5), range: 32–82 years; post-training group: *M* = 58.4 (*SD* = 12.2), range: 27–82 years **Cancer localization**: Brain, breast, gastrointestinal, head and neck, lung, urogenital, other tumors **Cancer grade**: N/A **Treatment**: Curative or palliative radiation therapy **Presence of a caregiver**: Yes (91% in the pre-training group, 89% in the post-training group) | Communicative behaviors Patients’ relative contribution to their consultation, contribution to agenda setting, provision of information about their ideas for diagnosis and prognosis, provision of psychosocial information, expressions of concern, biomedical questions, and contribution to decision-making | In the first 100 utterances, patients in the post-training group contributed a higher proportion to the interactions than patients in the pre-training group.  Overall, patients in the post-training group contributed about the same proportion of all utterances as patients in the pre-training group.  In the post-training group, patients discussed their psychosocial circumstances more, expressed more concerns, and asked more questions about their diagnosis.  Questions concerning general biomedical topics and prognoses were approximately the same in the pre- and post-training groups.  Patients in the post-training group discussed their treatment decision with more utterances and expressed their opinion on the treatment proposal with more utterances per consultation than those in the pre-training group. |
| [66] | Vos et al., 2022, Netherlands | N/A | Observational, qualitative, and quantitative study | Qualitative, with audio-recorded consultations and questionnaires | To describe colon cancer patients’ needs and how healthcare providers respond to these needs during routine follow-up consultations in hospital, as well as to assess patients’ satisfaction with information and communication, to gain comprehensive insights into the extent to which patients’ needs are addressed | **Participants**: Patients and providers  **Sample size**: *N* = 30  **Gender**: 50% female  **Age range**: *M* = 68,3  **Cancer localization**: Colon  **Cancer grade**: Stages I, II–III, and unknown  **Treatment**: Chemotherapy, unknown  **Presence of a caregiver**: No | Thematic analysis  Patients' behaviors | The 30 patients raised 120 questions, nearly all of which were categorized into the “health system and information” domain.  Most questions related to the planning of follow-up consultations and tests. Only a few questions were categorized into the “physical and daily living” domain.  In total, 420 cues and concerns were identified, of which 315 were patient-elicited, and 105 were healthcare provider-elicited. Most cues and concerns related to the “physical and daily living” domain, followed by the “health system and information” and “psychological” domains. Few cues and concerns were related to the “sexuality” domain.  The patients frequently reported symptoms related to colon cancer or its treatment. Some patients still found it difficult to resume daily activities and work. Cues and concerns in the “health system and information” domain often related to miscommunication or a lack of clarity about the follow-up schedule.  Some patients mentioned problems with their medication and side-effects, while others experienced difficulties with their insurance policies.  Patients also frequently expressed a fear of recurrence and the need for follow-up testing.  Some patients experienced social difficulties, while others reported diminished wellbeing and a loss of personal identity.  Few cues and concerns in the “sexuality” domain were expressed. One patient mentioned one, but the surgeon actively blocked further discussion of the problem. |
| [58] | Whisenant et al., 2021, USA | National Institutes of Health; National Institute of Nursing Research; the Hawn Foundation Fund for Education Programs in Pain and Symptom Research | Quantitative study | Quantitative, with audio-recorded consultations and questionnaires | To determine the extent to which women treated for breast cancer discuss their symptoms during the week prior to a clinic visit | **Participants**: Patients and providers  **Sample size**: *N* patients = 10; *N* providers = 50  **Gender**: 100% female (patients); 60% female (providers)  **Age range**: Patients: *M* = 51.6 (*SD* = 12.7), range: 24–70 years  **Cancer localization**: Breast  **Cancer grade**: Half II, half III, or IV  **Treatment**: Chemotherapy  **Presence of a caregiver**: N/A | Sociodemographic data  Symptom discussion  Time spent discussing each symptom | In the seven days prior to the 26 visits, there was a total of 157 calls to the automated symptom-reporting system across all participants, with an average of 5.80 calls. Participants provided 183 reports of moderate to severe symptoms. On average, the women experienced 7.04 moderate to severe symptoms during the week prior to a visit. Of the 101 moderate to severe symptoms reported, 50 (49.5%) were discussed at the next clinic visit, and 51 (50.5%) were not discussed.  Of the 101 reports of a moderate to severe symptom in the week prior to the clinic visit, the patient initiated 36% of symptom discussions, and the clinician initiated 64%.  Age impacted the likelihood of symptom discussion. Symptom discussions were more likely to occur among younger women: 60% of visits for women 40–49 years, 52.3% of visits for women 50–59 years, and 18.8% of visits for women 60 years or older.  The percentage of time spent discussing symptoms was similar across oncologist genders (29.9% for female oncologists and 24.5% for male oncologists). |
| [85] | Wollersheim et al., 2021, Netherlands | N/A | Observational and qualitative study | Qualitative, with audio-recorded consultations | To identify the nature of follow-up visits for prostate cancer survivors | **Participants**: Patients and providers **Sample size**: *N* = 32 **Gender**: 100% male (patients) **Age range**: N/A **Cancer localization:** Prostate **Cancer grade**: N/A **Treatment**: N/A **Presence of a caregiver**: N/A | Thematic analysis Patients’ expressions | The mean length of a follow-up consultation was 8 min and differed per consultation (range 3–14 min).  From the 32 follow-up visits, 119 cues and concerns and 144 questions were identified.  Most cues, concerns, and questions were related to the disease itself or the treatment of prostate cancer, while only 13 cases were not related to prostate cancer.  The most frequently expressed cues, concerns, and questions were reported in the health system and information domain, followed by the physical and daily living domain, psychological domain, and sexuality domain.  No cues, concerns, and questions were related to the patient care and support domain. |

** The results are taken directly from the leading articles and have been partially modified for greater readability.*
