# Supplementary material for: Exploring Communication Dynamics Between Patients and Healthcare Providers in Oncology: A Systematic Review
Source: Health Expect. 2025 Dec 11;28(6):e70519. doi: 10.1111/hex.70519 (PMC12696885; doi:10.1111/hex.70519)
Supplement: Supplementary file 2 — Supporting Information 2. [file HEX-28-e70519-s002.docx]

**Supplementary material 2.** Risk of bias assessment (MMAT).

| 1. **Qualitative studies** | | | | | | | | | | | | | | | | | | | | | | | | | | | | | | | | | | | | | |
| --- | --- | --- | --- | --- | --- | --- | --- | --- | --- | --- | --- | --- | --- | --- | --- | --- | --- | --- | --- | --- | --- | --- | --- | --- | --- | --- | --- | --- | --- | --- | --- | --- | --- | --- | --- | --- | --- |
| First author | | | | Year | | | | S1. Are there clear research questions? | | | | | S2. Do the collected data allow to address the research questions? | | | | | 1.1. Is the qualitative approach appropriate to answer the research question? | | | | | 1.2. Are the qualitative data collection methods adequate to address the research question? | | | | | 1.3. Are the findings adequately derived from the data? | | | | | 1.4. Is the interpretation of results sufficiently substantiated by data? | | | | 1.5. Is there coherence between qualitative data sources, collection, analysis and interpretation? |
| Amelung | | | | 2020 | | | | Yes | | | | | Yes | | | | | Yes | | | | | Yes | | | | | No | | | | | Yes | | | | Yes |
| Fagerlind | | | | 2008 | | | | Yes | | | | | Yes | | | | | Yes | | | | | Yes | | | | | Yes | | | | | Yes | | | | Yes |
| Fritz | | | | 2025 | | | | Yes | | | | | Yes | | | | | Yes | | | | | Yes | | | | | Yes | | | | | Yes | | | | Yes |
| Mitchell | | | | 2019 | | | | Yes | | | | | Yes | | | | | Yes | | | | | Yes | | | | | Yes | | | | | Yes | | | | Yes |
| Robinson | | | | 2016 | | | | Yes | | | | | Yes | | | | | Yes | | | | | Yes | | | | | Yes | | | | | Yes | | | | Yes |
| Rodriguez | | | | 2010 | | | | Yes | | | | | Yes | | | | | Yes | | | | | Yes | | | | | Yes | | | | | Yes | | | | Yes |
| Sherlock | | | | 2019 | | | | Yes | | | | | Yes | | | | | Yes | | | | | Yes | | | | | Yes | | | | | Yes | | | | Yes |
| Tang | | | | 2018 | | | | Yes | | | | | Yes | | | | | Yes | | | | | Yes | | | | | Yes | | | | | Yes | | | | Yes |
| Vos | | | | 2022 | | | | Yes | | | | | Yes | | | | | Yes | | | | | Yes | | | | | Yes | | | | | Yes | | | | Yes |
| Wollersheim | | | | 2021 | | | | Yes | | | | | Yes | | | | | Yes | | | | | Yes | | | | | Yes | | | | | Yes | | | | Yes |
| 1. **Randomized control trials** | | | | | | | | | | | | | | | | | | | | | | | | | | | | | | | | | | | | | |
| First author | | | Year | | | | | | S1. Are there clear research questions? | | | | | S2. Do the collected data allow to address the research questions? | | | | | 2.1. Is randomization appropriately performed? | | | | | 2.2. Are the groups comparable at baseline? | | | | | 2.3. Are there complete outcome data? | | | | | 2.4. Are outcome assessors blinded to the intervention provided? | | | 2.5 Did the participants adhere to the assigned intervention? |
| Bottacini | | | 2017 | | | | | | Yes | | | | | Yes | | | | | Yes | | | | | Yes | | | | | Yes | | | | | Yes | | | Yes |
| Buizza | | | 2020 | | | | | | Yes | | | | | Yes | | | | | Can't tell | | | | | Yes | | | | | Yes | | | | | Can't tell | | | Yes |
| Eggly | | | 2006 | | | | | | Yes | | | | | Yes | | | | | Yes | | | | | Yes | | | | | Yes | | | | | Can't tell | | | Yes |
| Eggly | | | 2017 | | | | | | Yes | | | | | Yes | | | | | Yes | | | | | Yes | | | | | Yes | | | | | Can't tell | | | Yes |
| Heyn | | | 2013 | | | | | | Yes | | | | | Yes | | | | | Can't tell | | | | | Yes | | | | | Yes | | | | | Yes | | | Yes |
| Ong | | | 2000 | | | | | | Yes | | | | | Yes | | | | | No | | | | | No | | | | | No | | | | | Can't tell | | | Yes |
| Song | | | 2015 | | | | | | Yes | | | | | Yes | | | | | Yes | | | | | Yes | | | | | No | | | | | Can't tell | | | Yes |
| Street | | | 2010 | | | | | | Yes | | | | | Yes | | | | | Yes | | | | | Yes | | | | | Yes | | | | | Yes | | | Yes |
| Street | | | 2014 | | | | | | Yes | | | | | Yes | | | | | No | | | | | Can't tell | | | | | No | | | | | No | | | Can't tell |
| Takeuchi | | | 2011 | | | | | | Yes | | | | | Yes | | | | | No | | | | | No | | | | | No | | | | | Can't tell | | | Can't tell |
| 1. **Non-randomized studies** | | | | | | | | | | | | | | | | | | | | | | | | | | | | | | | | | | | | | |
| First author | | Year | | | | S1. Are there clear research questions? | | | | | S2. Do the collected data allow to address the research questions? | | | | | 3.1. Are the participants representative of the target population? | | | | | 3.2. Are measurements appropriate regarding both the outcome and intervention (or exposure)? | | | | | 3.3. Are there complete outcome data? | | | | | 3.4. Are the confounders accounted for in the design and analysis? | | | | 3.5. During the study period, is the intervention administered (or exposure occurred) as intended? | | |
| Timmermans | | 2006 | | | | Yes | | | | | Yes | | | | | Yes | | | | | Yes | | | | | Yes | | | | | Can't tell | | | | Yes | | |
| 1. **Quantitative descriptive studies** | | | | | | | | | | | | | | | | | | | | | | | | | | | | | | | | | | | | | |
| First author | Year | | | | | | S1. Are there clear research questions? | | | | | S2. Do the collected data allow to address the research questions? | | | | | 4.1. Is the sampling strategy relevant to address the research question? | | | | | 4.2. Is the sample representative of the target population? | | | | | 4.3. Are the measurements appropriate? | | | | | 4.4. Is the risk of nonresponse bias low? | | | | 4.5. Is the statistical analysis appropriate to answer the research question? | |
| Butow | 2002 | | | | | | Yes | | | | | Yes | | | | | Yes | | | | | Yes | | | | | Yes | | | | | Can't tell | | | | Yes | |
| Consolandi | 2024 | | | | | | Yes | | | | | Yes | | | | | Yes | | | | | No | | | | | Yes | | | | | Yes | | | | Yes | |
| Del Piccolo | 2014 | | | | | | Yes | | | | | Yes | | | | | Yes | | | | | Yes | | | | | Yes | | | | | No | | | | Yes | |
| Hamel | 2021 | | | | | | Yes | | | | | Yes | | | | | No | | | | | Yes | | | | | Yes | | | | | Yes | | | | Yes | |
| Henry | 2015 | | | | | | Yes | | | | | Yes | | | | | Yes | | | | | No | | | | | Yes | | | | | Yes | | | | Yes | |
| Marino | 2023 | | | | | | Yes | | | | | Yes | | | | | Yes | | | | | No | | | | | Yes | | | | | Yes | | | | Yes | |
| Step | 2009 | | | | | | Yes | | | | | Yes | | | | | No | | | | | No | | | | | Yes | | | | | Can't tell | | | | Yes | |
| Street | 2008 | | | | | | Yes | | | | | Yes | | | | | Yes | | | | | Yes | | | | | Yes | | | | | Can't tell | | | | Yes | |
| Whisenant | 2021 | | | | | | Yes | | | | | Yes | | | | | No | | | | | No | | | | | Yes | | | | | Can't tell | | | | Yes | |
| 1. **Mixed methods studies** | | | | | | | | | | | | | | | | | | | | | | | | | | | | | | | | | | | | | |
| First author | | | | | Year | | | | | S1. Are there clear research questions? | | | | | S2. Do the collected data allow to address the research questions? | | | | | 5.1. Is there an adequate rationale for using a mixed methods design to address the research question? | | | | | 5.2. Are the different components of the study effectively integrated to answer the research question? | | | | | 5.3. Are the outputs of the integration of qualitative and quantitative components adequately interpreted? | | | | 5.4. Are divergences and inconsistencies between quantitative and qualitative results adequately addressed? | | | 5.5. Do the different components of the study adhere to the quality criteria of each tradition of the methods involved? |
| Alexander | | | | | 2012 | | | | | Yes | | | | | Yes | | | | | Yes | | | | | Yes | | | | | Yes | | | | Yes | | | No |
| Amundsen | | | | | 2018 | | | | | Yes | | | | | Yes | | | | | Yes | | | | | No | | | | | Yes | | | | Yes | | | Can't tell |
| Beach | | | | | 2015 | | | | | Yes | | | | | Yes | | | | | Yes | | | | | Yes | | | | | Yes | | | | Yes | | | Yes |
| D'Agostino | | | | | 2018 | | | | | Yes | | | | | Yes | | | | | No | | | | | Yes | | | | | Yes | | | | Yes | | | Can't tell |
| Del Piccolo | | | | | 2019 | | | | | Yes | | | | | Yes | | | | | No | | | | | Yes | | | | | Yes | | | | Yes | | | Yes |
| Eggly | | | | | 2015 | | | | | Yes | | | | | Yes | | | | | Yes | | | | | Yes | | | | | No | | | | Yes | | | No |
| Goss | | | | | 2015 | | | | | Yes | | | | | Yes | | | | | Yes | | | | | Yes | | | | | Yes | | | | Yes | | | Yes |
| Hack | | | | | 2010 | | | | | Yes | | | | | Yes | | | | | Yes | | | | | Yes | | | | | Yes | | | | Yes | | | Can't tell |
| Ishikawa | | | | | 2002a | | | | | Yes | | | | | Yes | | | | | Yes | | | | | Yes | | | | | Can't tell | | | | Yes | | | Can't tell |
| Ishikawa | | | | | 2002b | | | | | Yes | | | | | Yes | | | | | No | | | | | Yes | | | | | Yes | | | | Yes | | | Can't tell |
| Leppin | | | | | 2018 | | | | | Yes | | | | | Yes | | | | | No | | | | | Yes | | | | | Yes | | | | Yes | | | Can't tell |
| O'Neill | | | | | 2021 | | | | | Yes | | | | | Yes | | | | | No | | | | | Yes | | | | | Yes | | | | Yes | | | Yes |
| Reese | | | | | 2019 | | | | | Yes | | | | | Yes | | | | | No | | | | | Yes | | | | | Yes | | | | Yes | | | Yes |
| Roter | | | | | 2016 | | | | | Yes | | | | | Yes | | | | | Yes | | | | | Yes | | | | | Yes | | | | Yes | | | Can't tell |
| Schellenberger | | | | | 2022 | | | | | Yes | | | | | Yes | | | | | Yes | | | | | Yes | | | | | No | | | | Yes | | | Can't tell |
| Siminoff | | | | | 2000 | | | | | Yes | | | | | Yes | | | | | No | | | | | Yes | | | | | Yes | | | | Yes | | | Can't tell |
| Siminoff | | | | | 2006 | | | | | Yes | | | | | Yes | | | | | Can't tell | | | | | Yes | | | | | Yes | | | | Yes | | | Can't tell |
